# Synthesis and Properties of Boron Fluoride Complexes Using 2‑(*N*‑Pyridylamino)-1-azaazulene derivatives

**DOI:** 10.1021/acs.joc.5c02605

**Published:** 2026-03-23

**Authors:** Hibiki Morimoto, Tatsuya Iwashina, Kazuki Ohira, Yousuke Ooyama, Kazuki Yamamoto, Takahiro Gunji

**Affiliations:** 1 Department of Pure and Applied Chemistry, Faculty of Science and Technology, 26413 Tokyo University of Science, 2641 Yamazaki, Noda, Chiba 278-8510, Japan; 2 Program of Applied Chemistry, Graduate School of Advanced Science and Engineering, 12803 Hiroshima University, 1-4-1 Kagamiyama, Higashihiroshima, Hiroshima 739-8527, Japan

## Abstract

Three new boron fluoride complexes were synthesized using azaazulene derivatives, and their optical properties were investigated. These complexes were identified using single-crystal X-ray structural analysis, nuclear magnetic resonance spectroscopy, and Fourier transform infrared spectroscopy. We investigated the optical properties of these complexes in dichloromethane solution, poly­(styrene) films, and the solid state. The addition of acid led to a hypsochromic shift of approximately 60–90 nm in the absorption and fluorescence wavelengths in the dichloromethane solution and poly­(styrene) film. Moreover, two boron fluoride complexes exhibited up to 28.7-fold increases in fluorescence intensity upon protonation, while the remaining one exhibited fluorescence quenching. Density functional theory calculations revealed that the excited-state intramolecular proton transfer (ESIPT) involving the nitro group and subsequent donor excited photoinduced electron transfer (d-PeT) are responsible for these differences.

## Graphical abstract

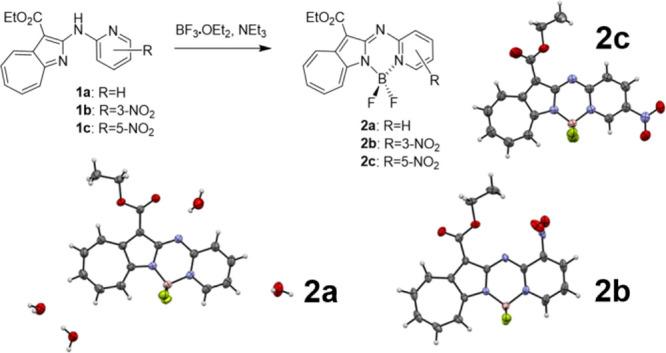

## 1 Introduction

Boron dipyrromethene (BODIPY) dyes were first discovered in 1968 by Treibs and Kreuzer.^1^ BODIPY is endowed with a high molar absorption coefficient, photoluminescence quantum yield, and chemical and optical stabilities;^2^ therefore, it is used in various applications, such as dye-sensitized solar cells^3,4^ and cell-imaging,^5^ chemical-sensing­[,^6–8^ and two-photon-imaging applications.^9,10^ There are many analogs of BODIPY, including aza-BODIPY,^11^ BODIHY,^12^ BOIMPY,^13^ BOIMPY,^14,15^ aza-BOIMPY,^16^ BOPHY,^17^ BOPPY,^18^ indigo-*N,N*’-diarylimine boron fluoride complexes,^19^ DIPYR,^20,21^ BTAA,^22^ and compound A^23^ (Figure 1). BODIPY, aza-BODIPY, BOIMPY, and aza-BOIMPY were constructed from two five-membered rings to categorize these boron fluoride complexes according to ring size. This type of complex, which is characterized by a simpler ligand structure, is less stable. While unsubstituted BODIPY was first synthesized in 2009 by Jung et al.^24^ and Peña-Cabrera et al.,^25^ unsubstituted aza-BODIPY is yet to be synthesized. DIPYR and BTAA, which contain two six-membered rings, are reportedly unstable to acid.^22^ Compound A, which contains a five-membered and a six-membered ring, reportedly exhibits a large Stokes shift,^23^ which may suppress the fluorescence decrease associated with self-absorption.

**Figure 1:**
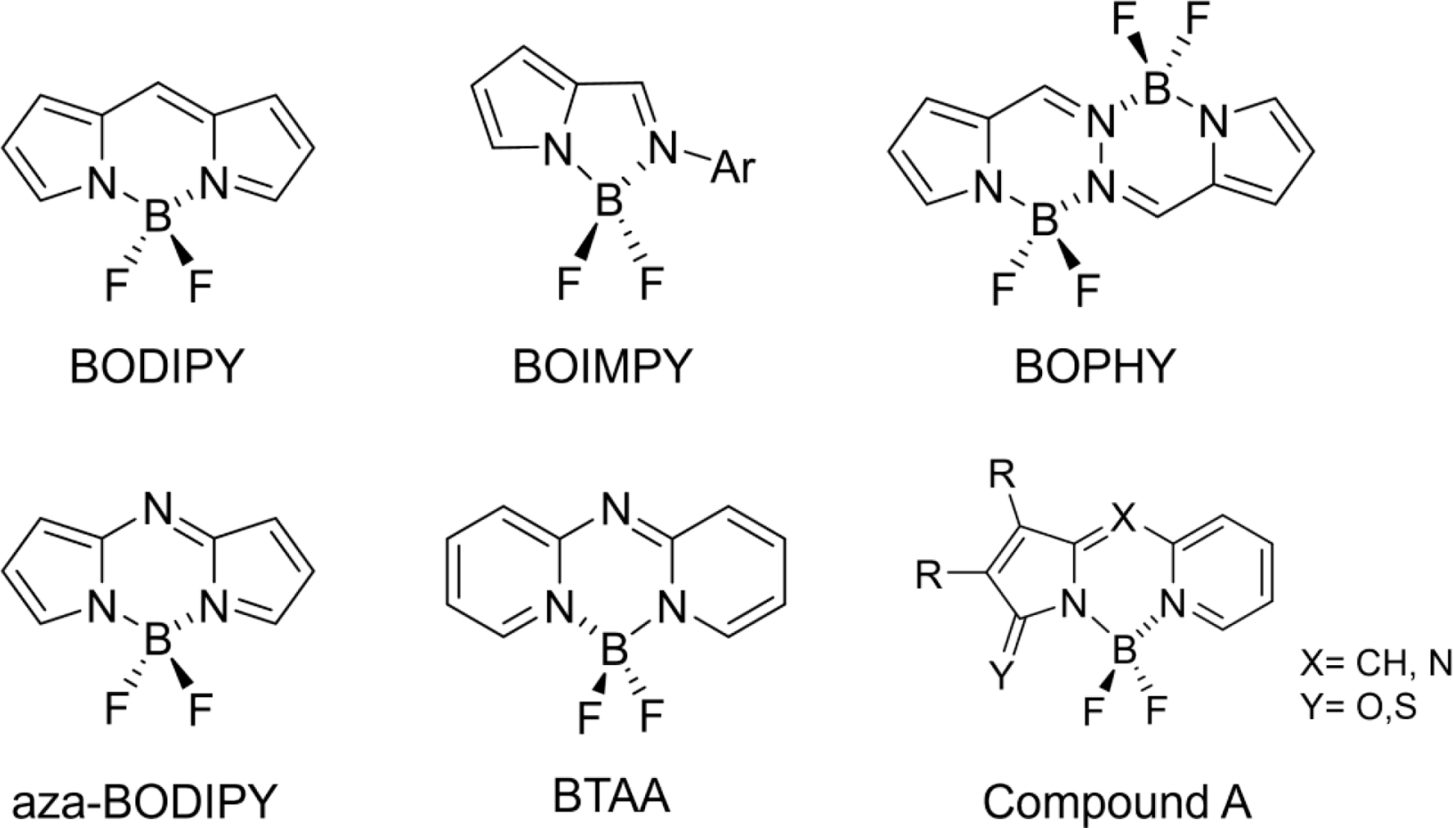
Structure of BODIPYs.

The construction of five- or six-membered-ring-type boron-fluoride complexes requires exocyclic double bonds. This problem is solved in compound A by the introduction of heteroatoms, such as oxygen and sulfur.^23^ Another way of satisfying the exocyclic double bond requirement involves the use of nonalternating conjugated aromatics, with azaazulene offering a stable and simple nonalternating conjugated aromatic structure.

Azaazulene is a typical nonalternating conjugated aromatic compound. Azaazulene has unique properties, including a large dipole moment of 3.05 D,^26^ and violates Kasha’s rule.^27^ Azaazulene derivatives have gained attention for their use in pharmacological­[ ^28–30^ and chemical-sensing^31,32^ applications, and have been explored theorically.^33,34^ However, the luminescence properties of azaazulenes are unclear and rarely studied.

In this study, we synthesized azaazulenes that are bridged to pyridines through nitrogen ligands and their boron difluoride complexes. The properties of five- and six-membered-ring-type boron fluoride complexes were investigated using 2-aminopyridine, which was chosen owing to its simple structure, and 2-amino-3-nitropyridine and 2-amino-5-nitropyridine, which were chosen because they have very different electronic states. The properties of these compounds and those of their protonated structures were examined using ultraviolet–visible (UV–Vis) spectroscopy, fluorescence spectroscopy, and density functional theory (DFT) calculations.

## 2 Results and Discussion

### 2.1 Synthesis

Compounds **1a**–**1c** were synthesized via Buchwald–Hartwig cross-coupling chemistry involving ethyl 2-chloro-1-azaazulene-3-carboxylate and various 2-pyridylamine analogs (Scheme 1). Among the studied aminopyridines, 2-amino-3-nitropyridine was found to be significantly more reactive than 2-aminopyridine or 2-amino-5-nitropyridine, with the former requiring 2 h for compete conversion, while the latter two required 22 h. Furthermore, the reaction of 2-amino-3-nitropyridine resulted in a mixture of **1b** and **1b’**, the double Buchwald–Hartwig cross-coupling product.^35,36^

**Scheme 1:**
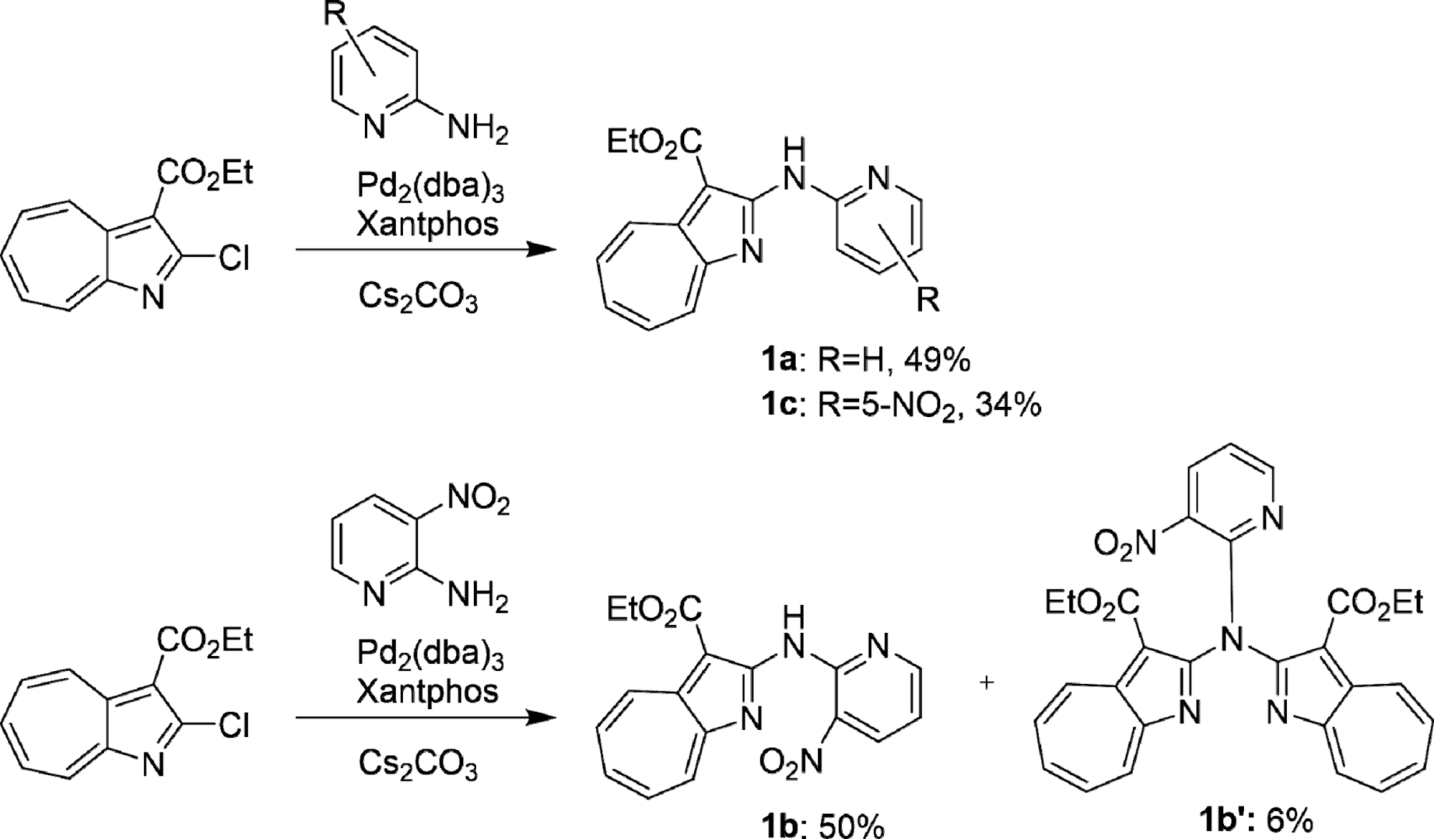
Synthesis of Compounds **1a**–**1c**

Compounds **2a**–**2c** were synthesized from **1a**–**1c**, boron trifluoride diethyl ether, and triethylamine (Scheme 2); here **1a** was observed to be less reactive than **1b** or **1c**, which required stirring in an ice bath for 30 min during reaction. In contrast, no product was formed when **1a** was reacted under these conditions, with refluxing in toluene for 2 h required to produce **2a**. These differences in reactivity are ascribable to the acidity of the cross-linking nitrogen. It was suggested that the nitro group in **1b** or **1c** improves the acidity of this nitrogen.

**Scheme 2:**
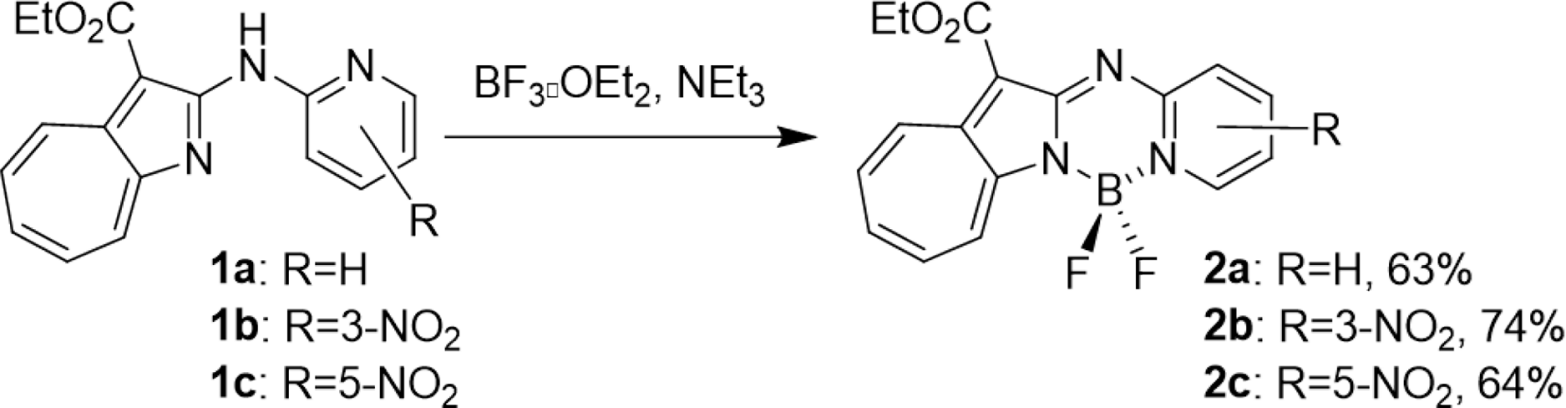
Synthesis of Compounds **2a**–**2c**

### 2.2 Single-Crystal X-ray Structural Analysis

Single-crystal X-ray diffractometry (XRD) was used to analyze the structures of **2a**–**2c**. Single crystals of **2a** suitable for XRD were obtained by the slow evaporation of nondehydrated acetone. It should be noted that XRD-suitable single crystals of **2a** were not obtained from dehydrated solvents. On the other hand, single crystals of **2b** were obtained by recrystallization from ethanol/hexane at different temperatures, while single crystals of **2c** were obtained by solvent diffusion using acetone and hexane. The XRD-determined crystal structures are shown in Figure 2, with corresponding data listed in Table S1. The crystal unit of **2a** contains four H_2_O molecules, with **2a** and H_2_O molecules exhibiting an alternating layered structure. The ester group in **2a** is configured oppositely to that in **2b** or **2c**, which is ascribable to hydrogen bonding. The crystal structure of **2b** is different to that of **2a** or **2c** (Figure S1) because the nitro group is unable to exist in the same place as the rest of the molecule due to steric and electronic hindrance.

**Figure 2:**
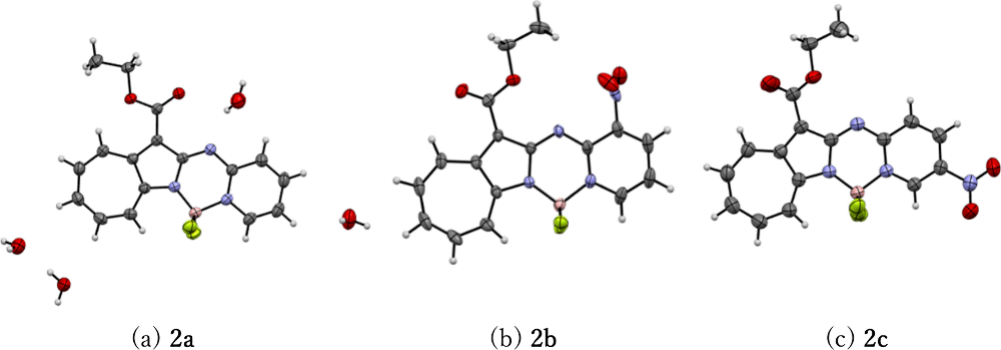
ORTEP drawings of (a) **2a**, (b) **2b**, and (c) **2c** with thermal ellipsoids drawn at the 50% probability level. Color code: white, H; pink, B; black, C; light blue, N; red, O; yellow green, F.

The angles between the long axes of **2a**–**2c** and their centerlines were determined to be 21.4°, 30.7°, and 19.6° respectively; therefore, these molecules form J-type aggregates.

### 2.3 Optical Properties

#### 2.3.1 UV–Vis and Fluorescence Spectra of **2a**–**2c**

Solutions of **2a**–**2c** in dichloromethane were subjected to UV–vis and fluorescence spectroscopy in order to study how the substituents affect the energy levels of these compounds, the results of which are shown in Figure 3; gas phase UV–vis and fluorescence spectra were also constructed using Time-Dependent Density Functional Theory (TDDFT) calculations.

**Figure 3:**
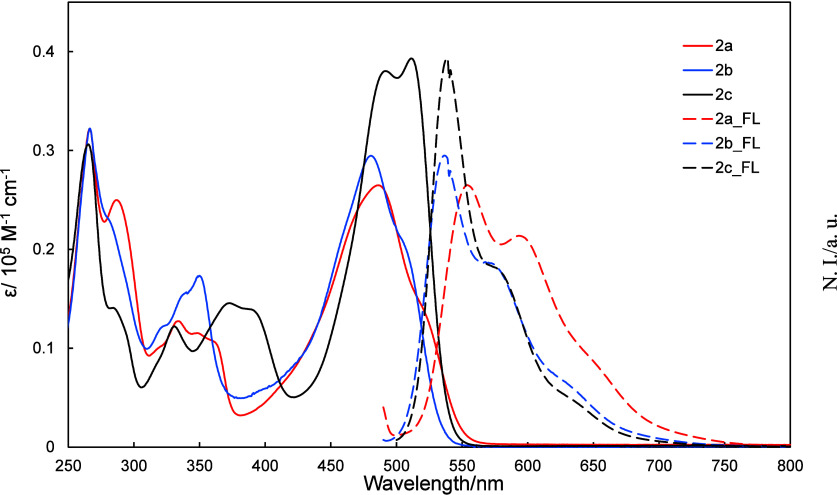
UV–vis and normalized fluorescence spectra of **2a**–**2c** in dichloromethane.

Fluorescence quantum yields (ϕ_F_) of 3%, 12%, 22% were determined for **2a**, **2b**, and **2c**, respectively. No fluorescence from the S_2_ state was observed, in violation of Kasha’s rule for azaazulenes. The TDDFT calculations reveal that the absorption bands near 500 nm and the fluorescence bands near 550 nm are associated with HOMO–LUMO transitions, while the absorption bands near 360 nm are attributable to HOMO–LUMO+1 transitions (Table S2).

#### 2.3.2 Protonation of **2a**–**2c**

Compounds **2a**–**2c** have β-ketoimine structures that are widely found in ligands;^37,38^ consequently, we believe that these compounds bind protons. UV–vis and fluorescence spectroscopy as well as TDDFT calculations were used to investigate their protonation dependences.

UV–vis spectra of **2a**–**2c** in dichloromethane were recorded as increasing amounts of trifluoroacetic acid (TFA) and triethylamine (TEA) were added, the results of which are shown in Figures 4–6. The UV–vis spectra of **2a**–**2c** exhibited hypsochromic shifts as TFA was added. The amount of TFA required to observed changes in these spectra depended on the molecule in question, with **2a**, **2b**, and **2c** requiring 20, 100, and 500 equiv., respectively. The protonation of **2a–2c** is due to the stabilization by the formation of tropylium cation.

**Figure 4:**
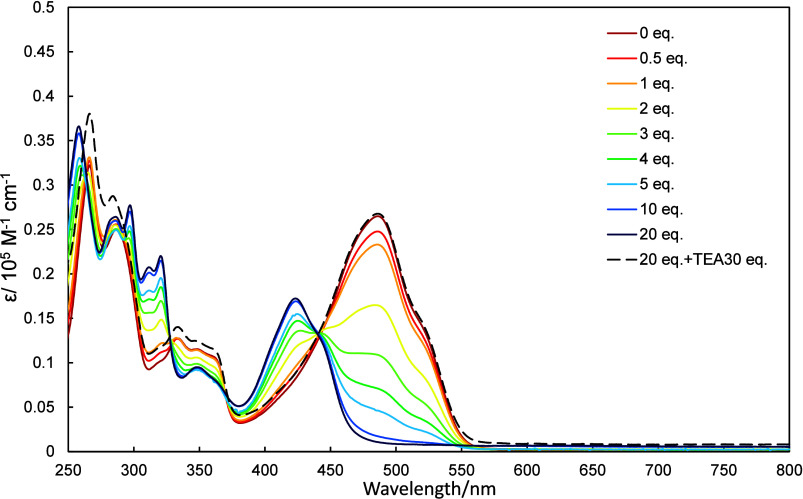
UV–vis spectra of **2a** (10 μmol/L in dichloromethane) recorded in the presence of various amounts of TFA (0–20 equiv) and then neutralized with 30 equiv of TEA.

**5:**
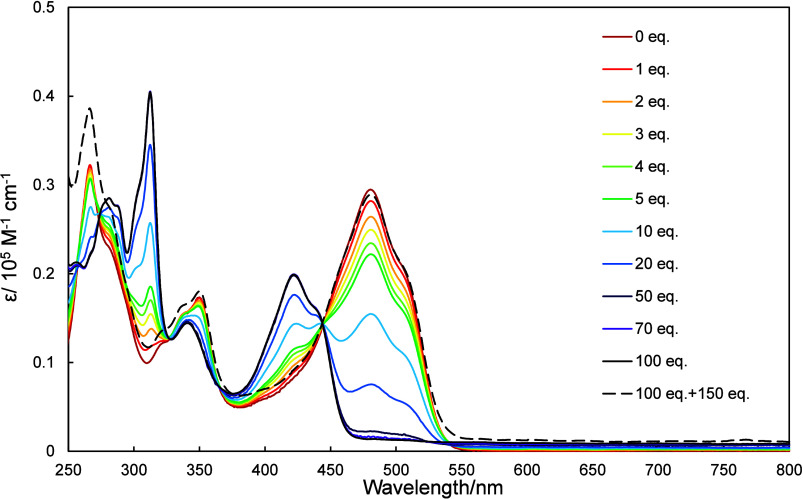
UV–vis spectra of **2b** (10 μmol/L in dichloromethane) recorded in the presence of various amounts of TFA (0–100 equiv) and then neutralized with 150 equiv of TEA.

**Figure 6:**
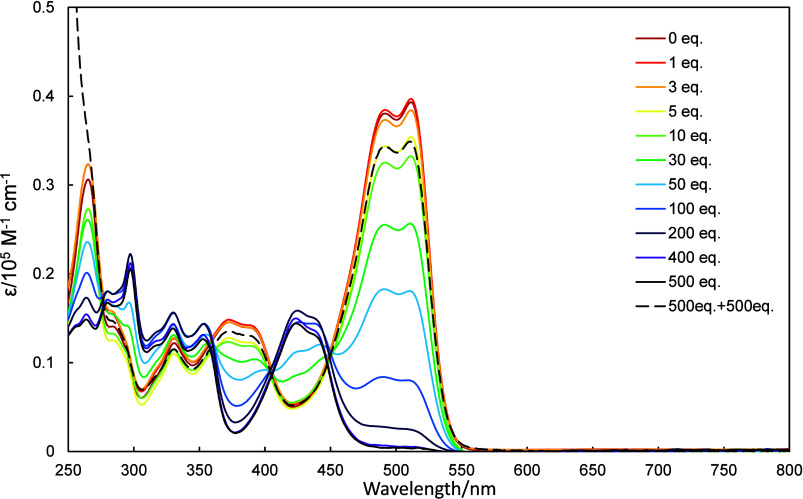
UV–vis spectra of **2c** (10 μmol/L in CH_2_Cl_2_) recorded in the presence of various amounts of TFA (0–500 equiv) and then neutralized with 500 equiv of TEA.

Fluorescence spectra of **2a**–**2c** acquired as different amount of TFA were added are shown in Figures 7–9; these spectra also show hypsochromic shifts in response to TFA. The fluorescence bands of **2a**–**2c** decreased in intensity until 20, 100, and 500 equiv of the TFA had been added, respectively (Figures S2–S4), while the intensities of new fluorescence bands concurrently increased. The fluorescence reverted to that of the corresponding neutral state when TEA was added.

**Figure 7:**
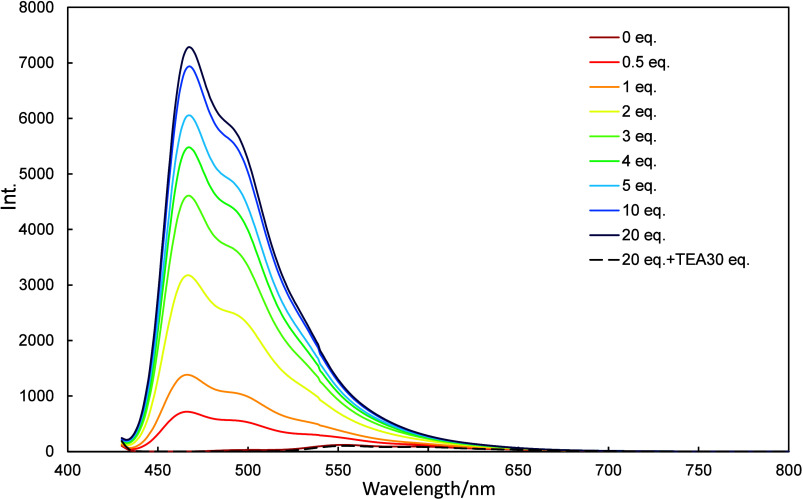
Fluorescence spectra of **2a** (10 μmol/L in dichloromethane) recorded in the presence of various amounts of TFA (0–20 equiv) and then neutralized with 30 equiv of TEA.

**8:**
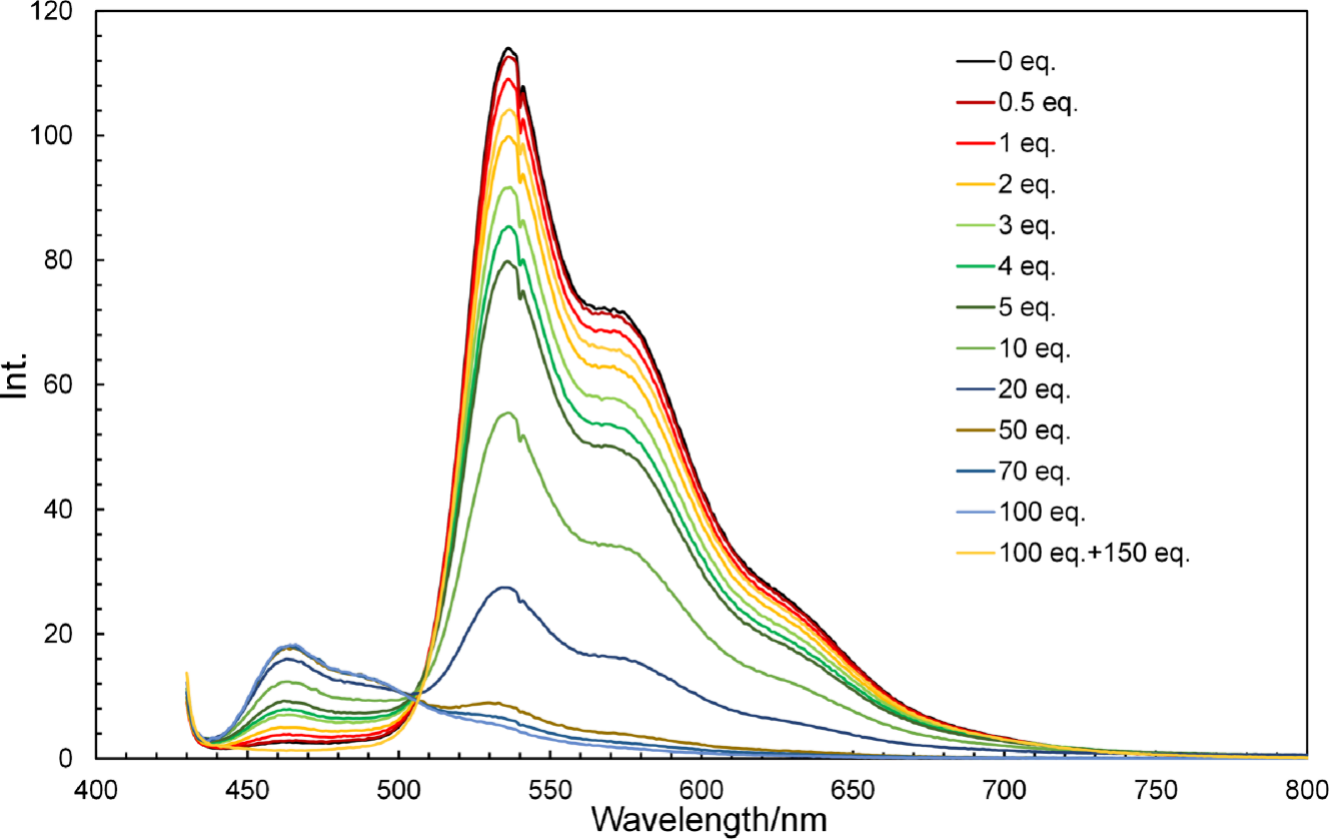
Fluorescence spectra of **2b** (10 μmol/L in dichloromethane) recorded in the presence of various amounts of TFA (0–100 equiv) and then neutralized with 150 equiv of TEA.

**Figure 9:**
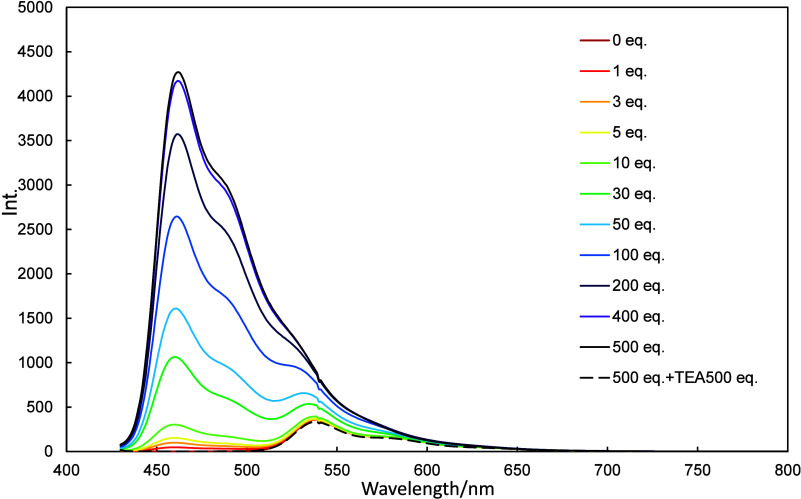
Fluorescence spectra of **2c** (10 μmol/L in dichloromethane) recorded in the presence of various amounts of TFA (0–500 equiv) and then neutralized with 500 equiv of TEA.

Intensity-normalized UV–vis and FL spectra are shown in Figure 10, and reveal that the three protonated species have similar spectra; **2a**+H^+^–**2c**+H^+^ absorbed most strongly in the visible region at 423, 422, and 423 nm, respectively, and fluoresced most strongly in the visible region at 467, 466, and 462 nm, respectively.

**Figure 10:**
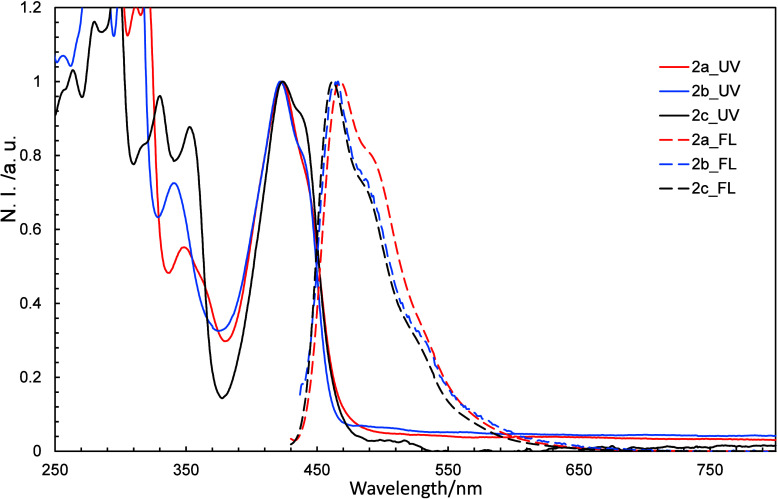
Normalized UV–vis and fluorescence spectra of **2a**–**2c** (10 μmol/L in dichloromethane).

The hypsochromic shifts associated with protonation were investigated by calculating the total energies of the ground and excited states, with protonation found to increase the energy gap between these two states (Figure 11). This observation was rationalized by calculating the differences in the electron densities of the HOMOs and LUMOs of the excited states of **2a** and **2a**+H^+^ (Figure 12). **2a** was found to be mainly excited over the entire molecule, whereas the azaazulene moiety in **2a**+H^+^ was mainly excited. Therefore, **2a** is excited in a localized manner by protonation, which increases the excitation energy. **2b**/**2b**+H^+^ and **2c**/**2c**+H^+^ exhibited similar trends (Figure S5). **2a**+H^+^–**2c**+H^+^ show excited-state HOMO–LUMO electron-density difference plots that are very similar in shape, which explains why **2a**+H^+^–**2c**+H^+^ exhibit very similar UV–vis and fluorescence bands.

**Figure 11:**
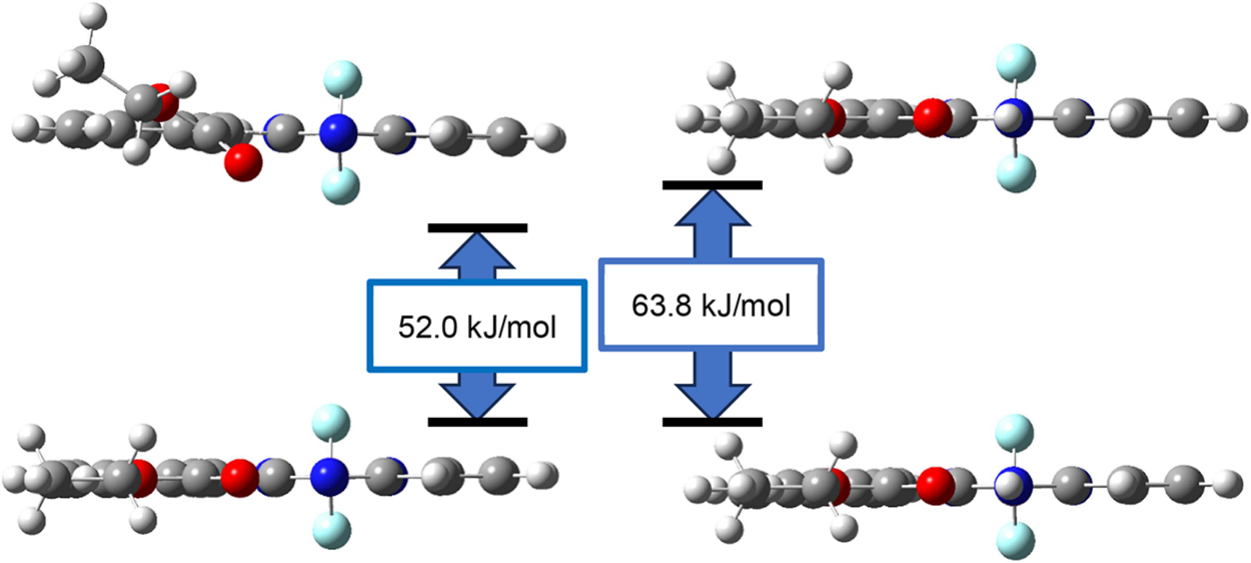
DFT-optimized structures of **2a** (left) and **2a**+H^+^ (right) in their ground (upper) and excited (lower) states, along with the calculated energy gaps.

**Figure 12:**
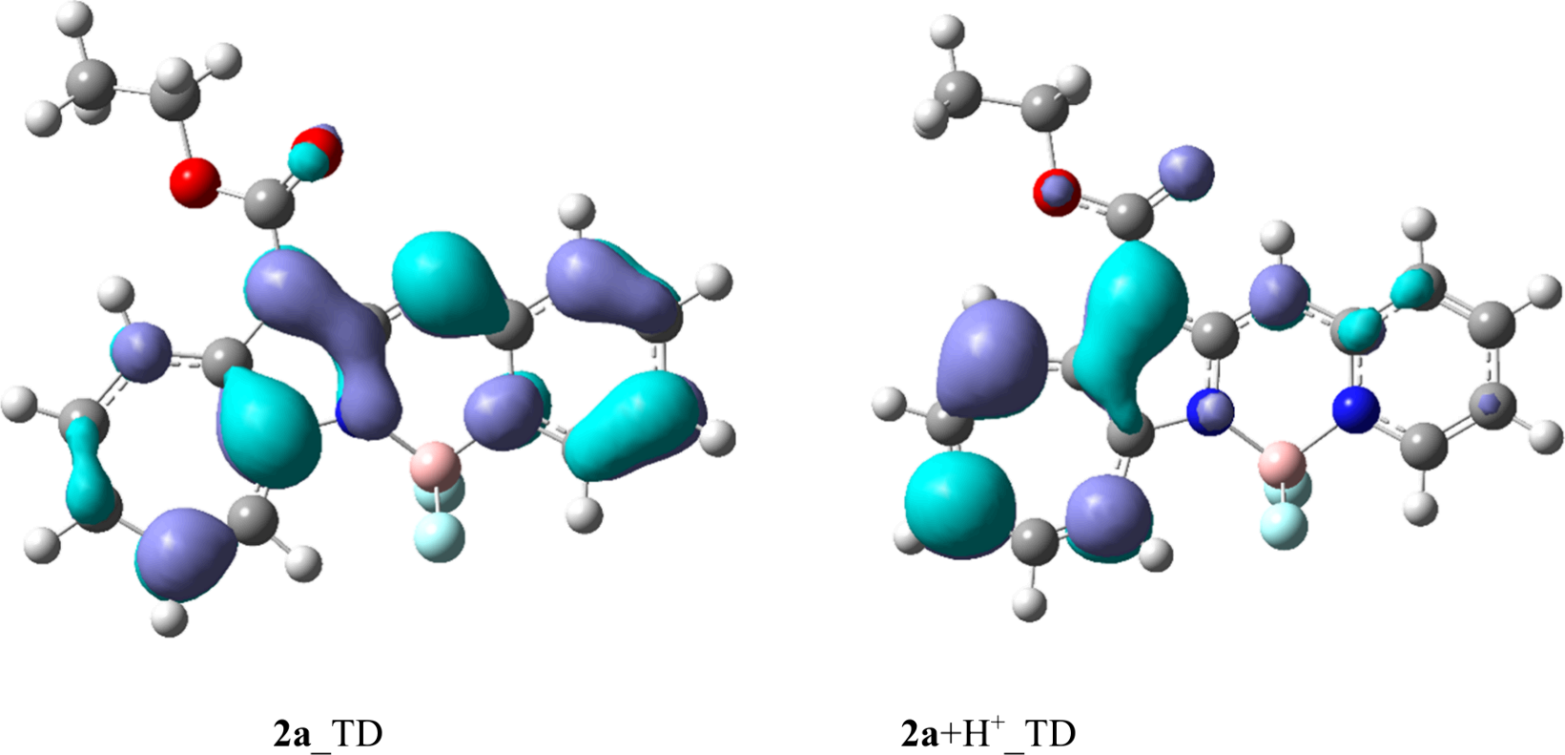
Electron density difference plots for **2a** (left) and **2a**+H^+^ (right). The light blue zones indicate electron density loss upon transition (donors), whereas the purple zones correspond to increases in electron density upon transition (acceptors).

**2a**+H^+^–**2c**+H^+^ exhibited a different fluorescence-intensity trend; **2a**/**2a**+H^+^, **2b**/**2b**+H^+^, and **2c**/**2c**+H^+^ exhibited intensity ratios of 28.7, 0.06, 2.57, respectively (Figure 13 and Table 1). **2a**+H^+^ and **2c**+H^+^ fluoresced more intensely compared to **2a** and **2c**. In contrast, **2b**+H^+^ fluoresced less intensely than **2b.**

**Figure 13:**
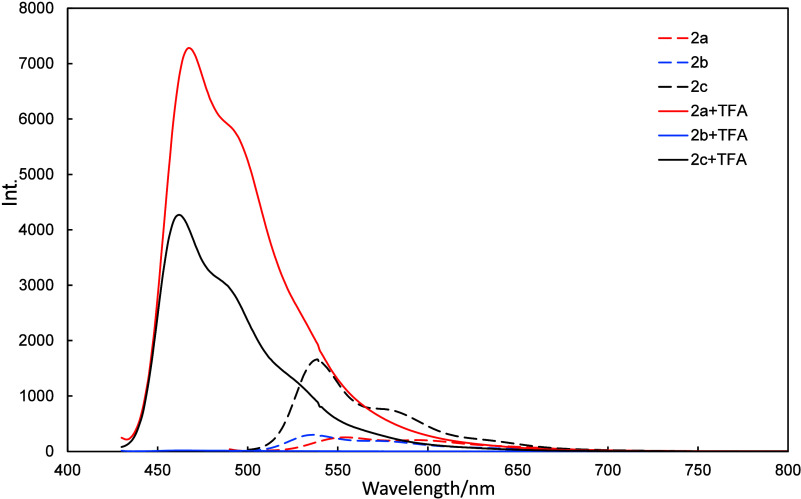
Fluorescence intensities of **2a**–**2c** and **2a**+H^+^–**2c**+H^+^.

**Table 1:** Fluorescence Intensities of **2a**–**2c** and **2a**+H^+^–**2c**+H^+^, and **2a**+H^+^/**2a**–**2c**+H^+^/**2c** Intensity Ratios

	neutral/nm			H^+^/ nm			
	λ_abs._	λ_FL_	FL int.	λ_abs._	λ_FL_	FL int.	int. ratio
**2a**	486	556	253	423	467	7281	28.7
**2b**	480	537	300	422	466	18	0.06
**2c**	511	538	1656	423	462	4270	2.57

The structures of the ground and excited states were optimized, which revealed that while the ester groups of **2a**–**2c** rotated upon excitation (Figure 11), those of **2a**+H^+^–**2c**+H^+^ did not, which is ascribable to hydrogen bonding between the hydrogen in the cross-link and the oxygen in the carbonyl group that prevents rotation of the ester group (Figure 11), thereby suppressing nonradiative decay and weakening fluorescence.

Compound **2b** exhibited a different trend from that observed for **2a** and **2c**; **2b** fluoresced less strongly with increasing acidity. The excited state of **2b**+H^+^ was structurally optimized, which revealed two stable structures: structure A, in which the added proton resides on the cross-linking nitrogen (Figure 14, left), and structure B, in which the proton is transferred to the nitro group (Figure 14, right). Structure B was calculated to be more stable than structure A by about 40 kJ/mol; consequently, the proton migrates to the nitro group when **2b**+H^+^ is excited; in other words, excited-state intramolecular proton transfer (ESIPT) occurs.

**Figure 14:**
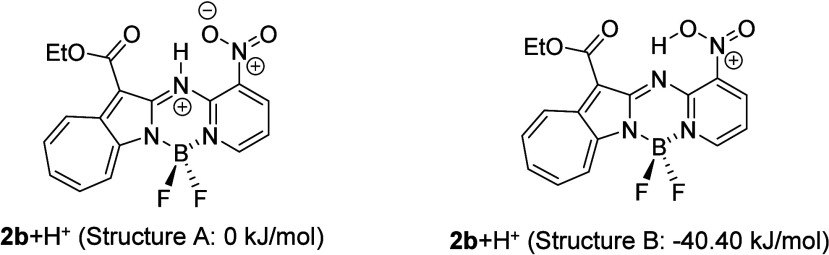
Added proton that resides on the cross-linking nitrogen (Structure A, left) migrates to the nitro group (Structure B, right) following excitation.

If fluorescent, structure B would be expected to exhibit a bathochromically shifted fluorescence wavelength owing to ESIPT leading to a lower energy state. However, **2a**+H^+^–**2c**+H^+^ exhibited very similar fluorescence wavelengths, with no longer-wavelength fluorescence observed (Figure 13). These results suggest that structure B deactivates in a nonradiative manner, with fluorescence only observed from structure A. However, structure B is more stable than structure A; therefore, most of the excited molecules are converted to the nonfluorescent structure via ESIPT, with only a small amount of fluorescence derived from the radiative inactivation of structure A ; consequently, only **2b**+H^+^ fluoresces less intensely when acid is added.

We compared the HOMOs and LUMOs of structures A and B in their excited states to elucidate the reason why structure B undergoes fluorescence quenching (Figure 15.). A comparison of the HOMOs of excited state structures A and B revealed no significant differences. In contrast, the LUMOs of excited state structures A and B are different; that in structure A is delocalized throughout the molecule, whereas that in structure B is localized on the pyridine side; hence the HOMO and LUMO in excited state structure B are spatially separated and donor-excited photoinduced electron transfer (d-PeT) from the azaazulene ring to the pyridine ring occurs, which rationalizes the observed fluorescence quenching, as summarized in Figure 16.

**Figure 15:**
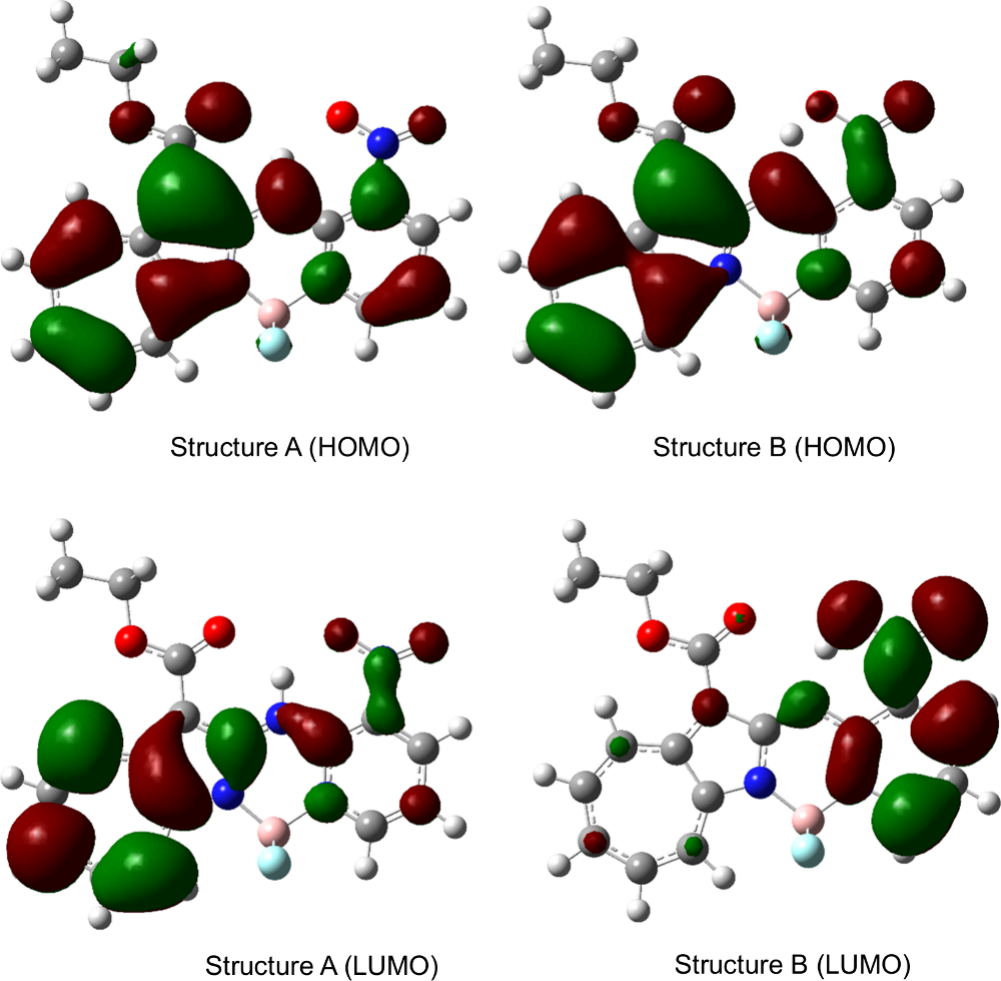
Orbital density distributions in **2b**+H^+^.

**Figure 16:**
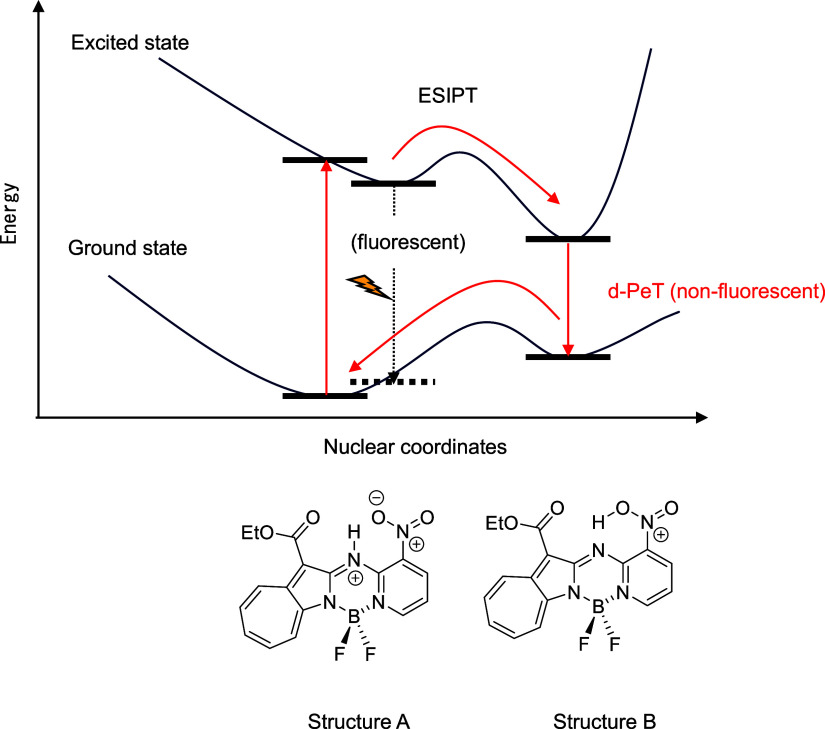
Energy diagram for **2b**+H^+^.

#### 2.3.3 Optical-Property Solvent Dependences

The solvent dependences of the absorption and fluorescence spectra of **2a**–**2c** were also investigated (Figures S6–S8), with no significant solvent-dependent wavelength changes observed; however, enhanced vibrational levels were observed with decreasing solvent polarity.

### 2.4 Solid State Properties of **2a**–**2c**

We also investigated the absorption and fluorescence properties of **2a**–**2c** in the solid state (Figure 17), which revealed bathochromic wavelengths shift in both the absorption and fluorescence spectra compared to those observed in the solution state, which is ascribable to the formation of J-aggregates, as discussed in Section 3.2.

**Figure 17:**
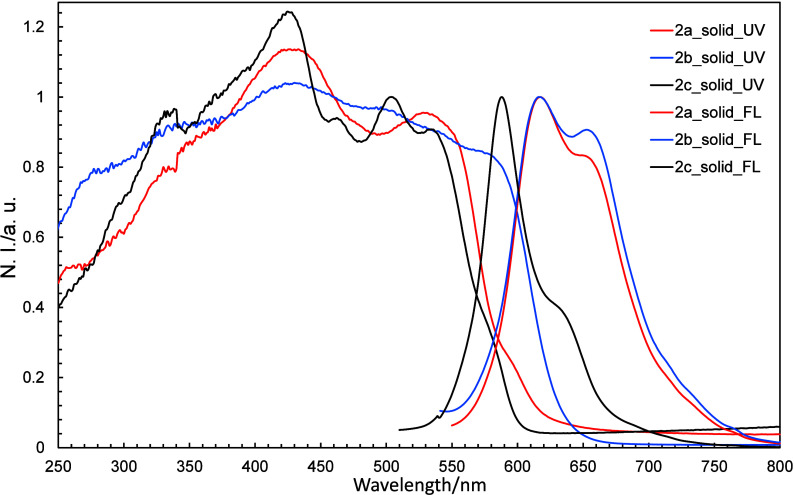
Normalized UV–vis and fluorescence spectra of **2a**–**2c** in the solid state.

### 2.5 Optical Properties of PS Films Containing **2a**–**2c**

PS films containing **2a**–**2c** were prepared to investigate the properties of the various solution and solid states. The method used to prepare the PS films containing **2a**–**2c** is shown in the SI. Similar UV–vis and fluorescence spectra to those acquired for the solutions were recorded when small amounts of **2a**–**2c** were added (Figure 18). Both the UV–vis and fluorescence spectra shifted to longer wavelengths with increasing amounts of **2a**–**2c** (Figure S9–S11).

**Figure 18:**
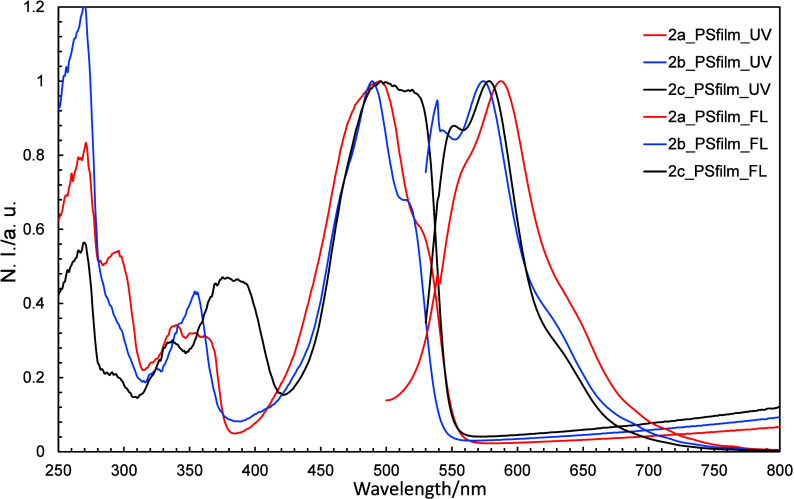
Normalized UV–vis and fluorescence spectra of **2a**–**2c** in PS films.

### 2.6 Optical Properties of PS Films Containing **2a**–**2c** and Added Acid

The method used to prepare PS films containing **2a**–**2c** is shown in the SI. Because significant spectral changes were observed as acid was added to the solution, we prepared PS films containing **2a**–**2c** and *p*-toluenesulfonic acid to determine whether or not similar protonation occurs in the PS films. *p*-Toluenesulfonic acid was selected because it is a strong solid acid. Figures 19 and 20 show UV–vis and fluorescence spectra of **2a**-based films, respectively, which reveal similar changes in the UV–vis and fluorescence spectra those observed for the solutions. In other words, protonation occurs in both the PS-film and solution states.

**Figure 19:**
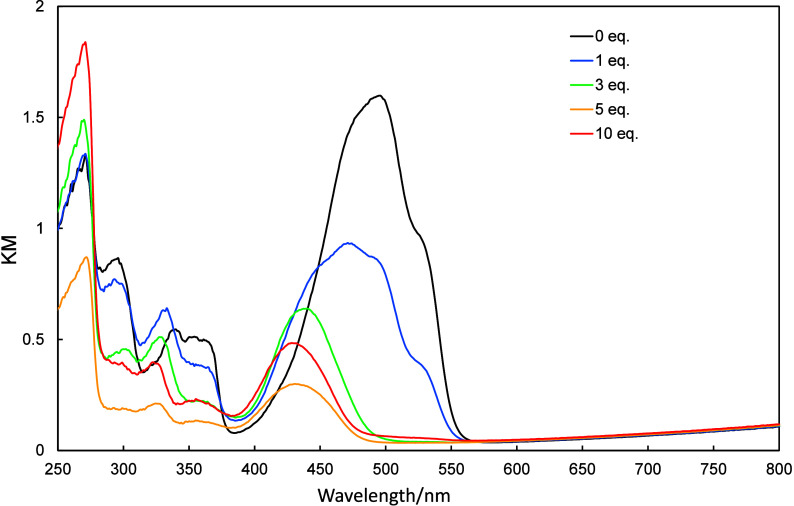
UV–vis spectra of **2a** in PS films containing *p*-toluenesulfonic acid.

**Figure 20:**
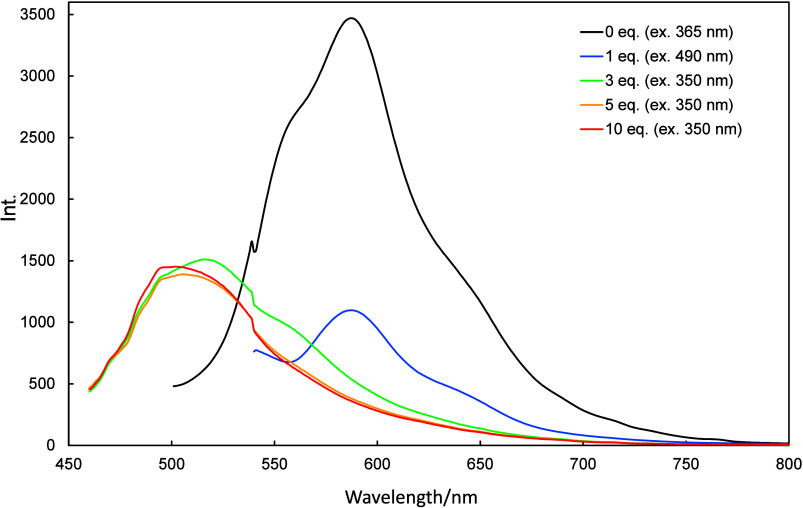
Fluorescence spectra of **2a** in PS films containing *p*-toluenesulfonic acid.

## 3 Conclusion

Compounds **2a**–**2c** were synthesized and their optical properties investigated. The UV–vis and fluorescence spectra of **2a**–**2c** exhibited bathochromic shifts in the order: CH_2_Cl_2_ solution < PS film < solid state. The absorption and fluorescence wavelengths of **2a**, **2b**, and **2c** were hypsochromic shifted by about 60–90 nm in both the CH_2_Cl_2_ and PS films upon the addition of acid, which is attributable to a change in the excitation range from whole-molecule to local excitation. Moreover, **2a** and **2c** fluoresced more strongly (by up to 28.7-fold) upon protonation. In contrast, **2b** exhibited fluorescence quenching ascribable to excited-state intramolecular proton transfer (ESIPT) via the nitro group and subsequent donor-excited photoinduced electron transfer (d-PeT).

## 4 Experimental Section

All chemical syntheses were performed under argon.

### 4.1 Materials

2-Aminopyridine, 2-amino-3-nitropyridine, 2-amino-5-nitropyridine, 4-amino-3-nitropyridine, tris­(dibenzylideneacetone)­dipalladium(0), xantphos, trifluoroacetic acid, and *p*-toluenesulfonic acid monohydrate were purchased from Tokyo Chemical Industry (Tokyo, Japan). Cesium carbonate, triethylamine, and boron trifluoride diethyl ether complex were purchased from FUJIFILM Wako Pure Chemical Corp. (Osaka, Japan). Polystyrene was purchased from Sigma–Aldrich (St. Louis, MO, USA). Silica-gel column chromatography was performed using Wakogel C-200 (spherical, neutral, 75–150 mm), while alumina column chromatography was performed using activated alumina (about 45 μm) purchased from FUJIFILM Wako Pure Chemical Corp. Ethyl 2-chloro-1-azaazulene-3-carboxylate (**1**) was prepared according to the literature,^28,39^ as detailed in the Supporting Information (SI).

### 4.2 Characterization

NMR spectra were recorded on a JEOL Resonance JNM-ECZ400S spectrometer (JEOL, Akishima, Japan) [^1^H at 400 MHz, ^13^C­{^1^H} at 100 MHz, and ^19^F at 376 MHz], a JNM-ECZ 400S spectrometer (JEOL, Akishima, Japan) [^1^H at 400 MHz, ^13^C­{^1^H} at 100 MHz, and ^19^F at 376 MHz], and a JNM-ECP 500 spectrometer (JEOL, Akishima, Japan) [^1^H at 500 MHz, ^13^C­{^1^H} at 125 MHz, and ^19^F at 470 MHz]. Chemical shifts are reported in ppm relative to chloroform-*d* (CDCl_3_) (^1^H residual chloroform peak at 7.26 ppm; ^13^C­{^1^H} peak at 77.16 ppm), DMSO-*d*_6_ (^1^H residual DMSO peak at 2.50 ppm; ^13^C­{^1^H} peak at 39.52 ppm), and hexafluorobenzene (^19^F = – 164.9 ppm) as an internal standard. High-resolution electrospray-ionization time-of-flight mass spectrometry (HR-ESI-TOF MS) was carried out on a JEOL JMS-T100CS “AccuTOF CS” spectrometer. UV–vis absorption spectra were recorded on a JASCO V-670 UV–vis-NIR spectrophotometer. Fourier-transform infrared (FT-IR) spectra were recorded on a JASCO FT/IR-6100 spectrometer (JASCO, Hachioji, Japan) using the ATR method. Elemental analyses was performed using a PerkinElmer 2400II Elemental Analyzer. Melting points were recorded using a Bibbly Stuart Scientific SMP3 instrument (reported melting points are uncorrected). Crystal data were collected using a XtaLAB Synergy-S diffractometer (RIGAKU, Japan) equipped with a monochromatic Mo–Kα radiation source (0.7107 Å). The CCDC deposition numbers for compounds **2a**–**c** are 2339627, 2339612, and 2339640, respectively. These data can be obtained free of charge at http://www.ccdc.cam.ac.uk/conts/re trieving.html, or from the Cambridge Crystallographic Data Centre, 12 Union Road, Cambridge CB2 1EZ, UK; fax: (+44) 1223–336–033; e-mail: deposit@ccdc.cam.ac.uk. The three-parameter Becke–Lee–Yang–Parr (B3LYP) hybrid exchange-correlation functional and the 6–31+G­(d,p) basis set (for H, C, O, N, B, and F) were used in our computational study. All calculations were performed using the Gaussian 16 W (Revision A.03) software program.^40^ Quantum yield was measured by HORIBA FluoroMax-4 spectrofluorometer with a calibrated integrating sphere system, and anthracene in ethanol was used as standards.

### 4.3 Synthesis of **1a**

Ethyl 2-chloro-1-azaazulene-3-carboxylate (0.50 g, 2.1 mmol), 2-aminopiridine (0.23 g, 2.5 mmol), xantphos (0.061 g, 0.11 mmol), tris­(dibenzylideneacetone)­dipalladium(0) (0.048 g, 0.053 mmol), cesium carbonate (0.96 g, 2.9 mmol), and dry 1,4-dioxane (12.5 mL) were mixed and refluxed by oil bath for 22 h, after which water (50 mL) was added. The resulting solution was extracted with chloroform, the organic layer was dried over Mg_2_SO_4_, filtered, and concentrated on a rotary evaporator. The residual solid was purified by silica-gel column chromatography (using dichloromethane then 1:1 (v/v) dichloromethane: ethyl acetate) and recrystallized from EtOH/hexane to afford a yellow solid (0.30 mg, 49%, lit.^28^ 72%).

**1a**: mp 131.5–134.2 °C. ^1^H NMR (CDCl_3_/400 MHz) δ (ppm) 10.22 (s, 1H), 9.07 (d, *J* = 10.4 Hz, 1H), 8.76 (d, *J* = 7.6 Hz, 1H), 8.45 (d, *J* = 7.6 Hz, 1H), 8.38 (d, *J* = 4.0 Hz, 1H), 7.78 (d, *J* = 10.4 Hz, 1H), 7.78 (d, *J* = 7.6 Hz, 1H), 7.74 (t, *J* = 9.6 Hz, 1H), 7.63 (t, *J* = 9.6 Hz, 1H), 6.99 (ddd, *J* = 0.8 Hz, 4.4 Hz, 8.0 Hz, 1H), 4.53 (q, *J* = 7.2 Hz, 2H), 1.52 (t, *J* = 7.2 Hz, 3H), ^13^C­{^1^H} NMR (CDCl_3_/100 MHz) δ (ppm) 165.7, 164.0, 160.5, 152.8, 147.0, 138.2, 134.0, 133.8, 133.5, 132.0, 131.6, 118.4, 113.6, 99.6, 60.5, 14.8. IR (ATR, cm^–1^): 3274 (νN–H), 2979 (νC–H), 1665 (νC = O). HR-ESI-MS *m*/*z* Calc. for C_17_H_15_N_3_O_2_Na: 316.1062 [M + Na]^+^; Found: 316.1060. Anal. Calc. for C_17_H_15_N_3_O_2_, 69.61; H, 5.15; N, 14.33. Found: C, 69.68; H, 5.05; N, 14.19.

### 4.4 Synthesis of **1b**

Ethyl 2-chloro-1-azaazulene-3-carboxylate (0.10 g, 0.42 mmol), 2-amino-3-nitropiridine (0.070 g, 0.50 mmol), xantphos (0.012 g, 0.021 mmol), tris­(dibenzylideneacetone)­dipalladium(0) (0.0097 g, 0.011 mmol), cesium carbonate (0.16 g, 0.59 mmol), and dry 1,4-dioxane (2.5 mL) were mixed and refluxed by oil bath for 2 h, after which water (10 mL) was added, and the solution was extracted using chloroform. The organic layer was dried over magnesium sulfate, filtered, and concentrated on a rotary evaporator. The residual solid was purified by silica-gel column chromatography (chloroform) and recrystallized from ethanol/hexane to afford brown crystals (73.4 mg, 50%).

**1b**: mp 175.4–176.2 °C. ^1^H NMR (CDCl_3_/400 MHz) δ (ppm) 12.59 (s, 1H), 9.32 (d, *J* = 10.0 Hz, 1H), 8.91 (dd, *J* = 1.6 Hz, 4.4 Hz, 1H), 8.76 (d, *J* = 8.8 Hz, 1H), 8.61 (dd, *J* = 1.6 Hz, 8.0 Hz, 1H), 7.79–7.91 (m, 3H), 7.16 (dd, *J* = 4.4 Hz, 8.0 Hz, 1H), 4.63 (q, *J* = 7.2 Hz, 2H), 1.54 (t, *J* = 7.2 Hz, 3H). ^13^C­{^1^H} NMR (CDCl_3_/100 MHz) δ (ppm) 165.3, 161.6, 159.4, 155.1, 147.1, 146.6, 136.5, 135.4, 135.2, 134.0, 133.8, 133.8, 132.3, 117.5, 102.0, 61.0, 14.9. IR (ATR, cm^–1^): 3204 (νN–H), 2976 (νC–H), 1666 (νC = O). Anal. Calc. for C_17_H_14_N_4_O_4_: C, 60.35; H, 4.17; N, 16.56. Found: C, 60.14; H, 3.95; N, 16.28.

### 4.5 Synthesis of **1c**

Ethyl 2-chloro-1-azaazulene-3-carboxylate (2.0 g, 8.4 mmol), 2-amino-5-nitropiridine (1.2 g, 8.5 mmol), xantphos (0.24 g, 0.42 mmol), tris­(dibenzylideneacetone)­dipalladium(0) (0.19 g, 0.21 mmol), cesium carbonate (3.3 g, 10 mmol), and dry 1,4-dioxane (50 mL) were mixed and refluxed by oil bath for 22 h, after which the solution was poured into water (100 mL) and filtered. The residue was washed with water, methanol, and chloroform to afford solid A. The filtrate was concentrated on a rotary evaporator and the residual solid was purified by silica-gel column chromatography (3:7 (v/v) ethyl acetate: hexane) to afford solid B. Solids A and B were combined and recrystallized from ethyl acetate to afford orange needles (0.98 g, 34%).

**1c**: mp 236.9–237.8 °C. ^1^H NMR (CDCl_3_/500 MHz) δ (ppm) 10.74 (s, 1H), 9.20 (s, 1H), 9.18 (d, *J* = 10.0 Hz, 1H), 9.05 (br, 1H), 8.55 (br, 1H), 8.51 (dd, *J* = 2.5 Hz, 10.0 Hz, 1H), 7.80–7.91 (m, 3H), 4.56 (q, *J* = 7.0 Hz, 2H), 1.54 (t, *J* = 7.0 Hz, 3H). ^13^C­{^1^H} NMR (CDCl_3_+TFA/100 MHz) δ (ppm) 154.3, 152.8, 145.2, 144.7, 143.2, 142.8, 141.1, 140.0, 139.5, 138.9, 135.0, 133.1, 113.8, 98.6, 63.2, 14.3; TFA: 114.8 (q, *J*_C–F_ = 142.3 Hz), 160.1 (q, *J*_C–F_ = 41.2 Hz). IR (ATR, cm^–1^): 3250 (νN–H), 2986 (νC–H), 1665 (νC = O). HR-ESI-MS *m*/*z* Calc. for C_17_H_14_N_4_O_4_Na: 361.0913 [M + Na]^+^; Found: 361.0906. Anal. Calc. for C_17_H_14_N_4_O_4_: C, 60.35; H, 4.17; N, 16.56. Found: C, 60.21; H, 4.01; N, 16.42.

### 4.6 Synthesis of **2a**

Compound **1a** (0.10 g, 0.34 mmol), dry toluene (20 mL), triethylamine (0.48 mL, 10 equiv), and boron trifluoride diethyl ether complex (0.58 mL, 15 equiv) were mixed and refluxed by oil bath for 2 h, after which water was added and the mixture was extracted with chloroform and dried over magnesium sulfate. The solution was filtered, concentrated on a rotary evaporator, and the residual solid was purified by silica-gel column chromatography (1:1 (v/v) ethyl acetate: hexane) and recrystallized from ethanol/hexane to afford a red solid (73.8 mg, 63%).

**2a**: mp 189.8–190.2 °C. ^1^H NMR (CDCl_3_/399 MHz) δ (ppm) 9.21 (d, *J* = 10.8 Hz, 1H), 8.47 (d, *J* = 10.0 Hz, 1H), 8.21 (d, *J* = 5.6 Hz, 1H), 7.81 (ddd, *J* = 1.6 Hz, 7.2 Hz, 8.8 Hz, 1H), 7.73 (t, *J* = 10.8 Hz, 2H), 7.58 (t, *J* = 10.0 Hz, 1H), 7.51 (d, *J* = 8.8 Hz, 1H), 7.03 (dt, *J* = 1.6 Hz, 5.6 Hz, 1H), 4.53 (q, *J* = 7.2 Hz, 2H), 1.50 (t, *J* = 7.2 Hz, 3H). ^13^C­{^1^H} NMR (CDCl_3_/125 MHz) δ (ppm) 164.1, 159.0, 155.0, 148.9, 147.8, 140.9, 137.5, 136.2, 135.5, 135.2, 133.5, 125.5, 124.3, 115.7, 105.1, 60.8, 14.6. ^19^F NMR (CDCl_3_/375 MHz) δ­(ppm) – 141.8 (q, *J* = 29.6 Hz, 2F). IR (ATR, cm^–1^): 3204 (νN–H), 2978 (νC–H), 1774 (νC = O). HR-ESI-MS *m*/*z* Calc. for C_17_H_14_N_3_O_2_BF_2_Na: 364.1048 [M + Na]^+^; Found: 364.1042. Anal. Calc. for C_17_H_14_N_3_O_2_BF_2_, 59.86; H, 4.14; N, 12.32. Found: C, 59.87; H, 3.90; N, 12.34.

### 4.7 Synthesis of **2b**

Compound **1b** (0.10 g, 0.29 mmol) and dry dichloromethane (20 mL) were mixed and cooled in an ice bath. Triethylamine (0.40 mL, 10 equiv) was added, after which boron trifluoride diethyl ether complex (0.49 mL, 15 equiv) was slowly added. The mixture was stirred for 30 min, after which 0.2 M aqueous sodium carbonate was added, and the mixture was stirred until the solid had dissolved. The solution was then extracted with dichloromethane, dried over magnesium sulfate, filtered, and concentrated on a rotary evaporator. The residual solid was purified by silica-gel column chromatography (1:2 (v/v) ethyl acetate: hexane) and recrystallized from ethanol/hexane to afford rhomboidal orange crystals (84.3 mg, 74%).

**2b**: mp 210.3–210.8 °C. ^1^H NMR (DMSO-*d*_6_/399 MHz) δ (ppm) 9.28 (d, *J* = 10.4 Hz, 1H), 8.57 (d, *J* = 7.6 Hz, 1H), 8.55 (d, *J* = 9.6 Hz, 1H), 8.48 (d, *J* = 6.4 Hz, 1H), 8.13 (t, *J* = 10.4 Hz, 1H), 8.11 (t, *J* = 10.4 Hz, 1H), 7.99 (t, *J* = 9.6 Hz, 1H), 7.25 (dd, *J* = 6.4 Hz, 7.6 Hz, 1H), 4.28 (q, *J* = 7.2 Hz, 2H), 1.38 (t, *J* = 7.2 Hz, 3H). ^13^C­{^1^H} NMR (DMSO-*d*_6_/100 MHz) δ (ppm) 163.2, 157.0, 147.2, 147.2, 146.7, 142.5, 140.9, 138.4, 138.0, 136.9, 135.7, 134.2, 127.4, 114.2, 103.9, 59.9, 13.8. ^19^F NMR (DMSO-*d*_6_/376 MHz) δ (ppm) – 137.3 (q, *J* = 30.0 Hz, 2F). IR (cm^–1^) (ATR): 3086 (νC–H), 1777 (νC = O). HR-ESI-MS *m*/*z* Calc. for C_17_H_13_N_4_O_4_ BF_2_Na: 409.0899 [M + Na]^+^; Found: 409.0886. Anal. Calc. for C_17_H_13_N_4_O_4_ BF_2_, 52.88; H, 3.39; N, 14.51. Found: C, 52.81; H, 3.08; N, 14.42.

### 4.8 Synthesis of **2c**

Compound **1c** (0.50 g, 1.4 mmol) and dry dichloromethane (200 mL) were mixed and cooled in an ice bath. Triethylamine (2.0 mL, 10 equiv) was added, after which boron trifluoride diethyl ether complex (2.45 mL, 15 equiv) was slowly added. The mixture was stirred for 30 min, after which water (100 mL) was added. The mixture was extracted with dichloromethane, dried over magnesium sulfate, filtered, and concentrated on a rotary evaporator. The residual solid was purified by alumina column chromatography (using 1:2 then 2:3 (v/v) ethyl acetate: hexane) and recrystallized from chloroform/ethanol to afford red crystals (0.36 g, 64%).

**2c**: mp 242.5–243.3 °C. ^1^H NMR (CDCl_3_/399 MHz) δ (ppm) 9.44 (d, *J* = 10.8 Hz, 1H), 9.15 (s, 1H), 8.74 (d, *J* = 9.6 Hz, 1H), 8.37 (dd, *J* = 2.8 Hz, 9.6 Hz, 1H), 7.96 (t, *J* = 9.6 Hz, 2H), 7.38 (d, *J* = 9.6 Hz, 1H), 4.54 (q, *J* = 7.2 Hz, 2H), 1.51 (t, *J* = 7.2 Hz, 3H). ^13^C­{^1^H} NMR (CDCl_3_/100 MHz) δ (ppm) 163.5, 158.4, 155.8, 148.0, 147.4, 138.3, 137.7, 137.1, 137.0, 136.3, 136.2, 133.5, 129.0, 124.2, 106.0, 61.2, 14.5, ^19^F NMR (CDCl_3_/375 MHz) δ (ppm) – 139.6 (q, *J*_B–F_ = 30.0 Hz, 2F). IR (cm^–1^) (ATR): 2987 (νC–H), 1787 (νC = O). HR-ESI-MS *m*/*z* Calc. for C_17_H_13_N_4_O_4_ BF_2_Na: 409.0899 [M + Na]^+^; Found: 409.0896. Anal. Calc. for C_17_H_13_N_4_O_4_ BF_2_, 52.88; H, 3.39; N, 14.51. Found: C, 52.98; H, 3.06; N, 14.59.

## Supplementary Material



## Data Availability

The data underlying this study are available in the published article and its Supporting Information.

## Notes

Experimental details, NMR, UV–Vis, fluorescence spectra, single crystal X-ray structural analysis, and DFT calculations (PDF)
