# Supplementary material for: Synthesis and Properties of Boron Fluoride Complexes Using 2‑(N‑Pyridylamino)-1-azaazulene derivatives
Source: J Org Chem. 2026 Mar 23;91(13):4559–71. doi: 10.1021/acs.joc.5c02605 (PMC13054864; doi:10.1021/acs.joc.5c02605)
Supplement: Supplementary file 1 [file jo5c02605_si_001.pdf]

Supporting information

Synthesis and Properties of Boron Fluoride Complexes Using 2-(*N*-Pyridylamino)-1-azaazulene derivatives

Hibiki Morimoto,[1] Tatsuya Iwashina,[1] Kazuki Ohira,[2] Yousuke Ooyama,[2] Kazuki Yamamoto,[1] and Takahiro Gunji*[1]

[1]Department of Pure and Applied Chemistry, Faculty of Science and Technology, Tokyo University of Science, 2641 Yamazaki, Noda, Chiba 278-8510, Japan

[2]Program of Applied Chemistry, Graduate School of Advanced Science and Engineering, Hiroshima University, 1-4-1 Kagamiyama, Higashihiroshima, Hiroshima 739-8527, Japan

Table of Contents

Table S1. Crystal data and structure refinement for **2a**, **2b**, **2c** S1

Fig. S1 Crystal structure of **2a**–**2c**. S1

Fig. S2 Fluorescence spectra of **2a** (ex. 485 nm). S26

Fig. S3 Fluorescence spectra of **2b** (ex. 480 nm). S26

Fig. S4 Fluorescence spectra of **2c** (ex. 490 nm). S27

Fig. S5 Electron density difference plots for **2a** (upper left), **2a**+H^+ (upper right), **2b** (center left), **2b**+H^+ (center right), **2c** (lower left), and **2c**+H^+ (lower right). The light blue zones indicate electron density loss upon transition (donor) whereas the purple zones correspond to increase of electron density upon transition (acceptors). S28

Fig. S6 Solvent dependence of **2a**................................ S29

Fig. S7 Solvent dependence of **2b**. S29

Fig. S8 Solvent dependence of **2c**. S30

Fig. S9 UV–vis and fluorescence spectra of PS films added with **2a**. S31

Fig. S10 UV–vis and fluorescence spectra of PS films added with **2b**. S31

Fig. S11 UV–vis and fluorescence spectra of PS films added with **2c**. S32

Fig. S12–S27 spectra data of **1a**–**2c**................................ S40

DFT calculation. S41–S63

Experimental Section. S64–S67

Table S1. Crystal data and structure refinement for **2a**, **2b**, **2c**

	**2a**	**2b**	**2c**
Empirical formula	$C_{17}H_{13}BF_2N_3O_2$, 4(H_2O)	1.333($C_{17}H_{13}BF_2N_4O_4$)	1.333($C_{17}H_{13}BF_2N_4O_4$)
Formula weight	413.18	514.83	514.83
Crystal system	Triclinic	Monoclinic	Monoclinic
Space group	P-1	P1 2_1/n1	P2_1/n
a / Å	8.7592(2)	12.6488(5)	9.7139(3)
b / Å	8.7707(3)	8.7874(3)	18.4738(4)
c / Å	13.1625(5)	14.8750(6)	10.0865(4)
V /$Å^3$	947.51(6)	1635.51(11)	1674.82(10)
α/deg	86.157(3)°	90°	90°
β/deg	78.971(3)°	98.426(4)°	112.288(4)°
γ/deg	72.689(3)°	90°	90°
GooF	1.070	1.030	1.077
R_1	0.0397	0.0455	0.0395
wR_2	0.1026	0.1209	0.1175
Z	2	3	3
CCDC	2339627	2339612	2339640

2a

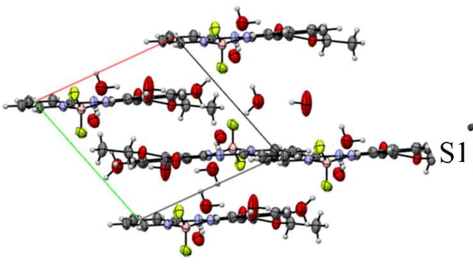

2b

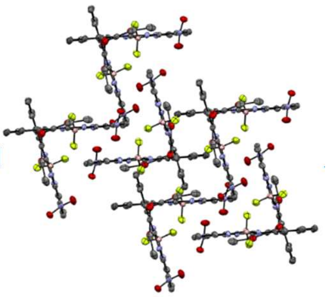

2c

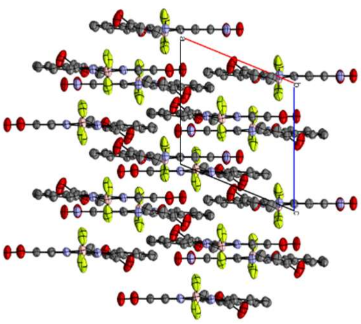

Table S2. Bond lengths of **2a**

Number	Atom1	Atom2	Length/ nm	Number	Atom1	Atom2	Length/ nm
1	F2	B1	1.384(2)	26	C1	C2	1.355(2)
2	F1	B1	1.380(2)	27	C10	H10	0.930
3	O2	C15	1.332(2)	28	C10	C11	1.399(2)
4	O2	C16	1.454(2)	29	C4	H4	0.930
5	O1	C15	1.216(2)	30	C4	C3	1.364(3)
6	N3	C14	1.380(2)	31	C16	H16A	0.970
7	N3	C6	1.379(2)	32	C16	H16B	0.970
8	N3	B1	1.530(2)	33	C16	C17	1.498(3)
9	N1	C5	1.364(2)	34	C12	H12	0.930
10	N1	C1	1.366(2)	35	C12	C11	1.366(2)
11	N1	B1	1.567(2)	36	C11	H11	0.930
12	N2	C6	1.334(2)	37	C2	H2	0.930
13	N2	C5	1.359(2)	38	C2	C3	1.392(2)
14	C7	C8	1.408(2)	39	C3	H3	0.930
15	C7	C6	1.427(2)	40	C17	H17A	0.960
16	C7	C15	1.459(2)	41	C17	H17B	0.960
17	C14	C8	1.453(2)	42	C17	H17C	0.960
18	C14	C13	1.381(2)	43	O3	H3A	0.84(2)
19	C8	C9	1.411(2)	44	O3	H3B	0.94(3)
20	C5	C4	1.415(2)	45	O6	H6A	0.93(3)
21	C13	H13	0.930	46	O6	H6B	0.90(2)
22	C13	C12	1.398(2)	47	O4	H4A	0.88(3)
23	C9	H9	0.930	48	O4	H4B	0.88(3)
24	C9	C10	1.373(2)	49	O5	H5A	0.85(3)
25	C1	H1	0.930	50	O5	H5B	0.85(3)

Table S3. Bond angles of **2a**

Number	Atom1	Atom2	Atom3	Angle/ °
1	C15	O2	C16	118.0(1)
2	C14	N3	C6	109.5(1)
3	C14	N3	B1	126.8(1)
4	C6	N3	B1	122.6(1)
5	C5	N1	C1	120.4(1)
6	C5	N1	B1	123.0(1)
7	C1	N1	B1	116.6(1)
8	C6	N2	C5	118.8(1)
9	C8	C7	C6	107.6(1)
10	C8	C7	C15	128.7(1)
11	C6	C7	C15	123.8(1)
12	N3	C14	C8	107.9(1)
13	N3	C14	C13	121.8(1)
14	C8	C14	C13	130.3(1)
15	C7	C8	C14	106.6(1)
16	C7	C8	C9	128.5(1)
17	C14	C8	C9	124.9(1)
18	N3	C6	N2	123.9(1)
19	N3	C6	C7	108.5(1)
20	N2	C6	C7	127.6(1)
21	N1	C5	N2	123.3(1)
22	N1	C5	C4	118.0(1)
23	N2	C5	C4	118.7(1)
24	O2	C15	O1	122.7(1)
25	O2	C15	C7	112.6(1)
26	O1	C15	C7	124.7(1)
27	C14	C13	H13	116.0
28	C14	C13	C12	127.9(1)
29	H13	C13	C12	116.0
30	C8	C9	H9	115.5
31	C8	C9	C10	129.1(1)

Number	Atom1	Atom2	Atom3	Angle/ °
32	H9	C9	C10	115.4
33	N1	C1	H1	118.7
34	N1	C1	C2	122.5(1)
35	H1	C1	C2	118.7
36	C9	C10	H10	114.9
37	C9	C10	C11	130.2(1)
38	H10	C10	C11	114.9
39	C5	C4	H4	119.7
40	C5	C4	C3	120.6(1)
41	H4	C4	C3	119.7
42	O2	C16	H16A	110.3
43	O2	C16	H16B	110.3
44	O2	C16	C17	106.9(1)
45	H16A	C16	H16B	108.6
46	H16A	C16	C17	110.4
47	H16B	C16	C17	110.3
48	C13	C12	H12	115.4
49	C13	C12	C11	129.2(1)
50	H12	C12	C11	115.4
51	C10	C11	C12	128.3(1)
52	C10	C11	H11	115.8
53	C12	C11	H11	115.8
54	C1	C2	H2	120.9
55	C1	C2	C3	118.2(1)
56	H2	C2	C3	120.9
57	C4	C3	C2	120.2(1)
58	C4	C3	H3	119.9
59	C2	C3	H3	119.9
60	C16	C17	H17A	109.5
61	C16	C17	H17B	109.5
62	C16	C17	H17C	109.5
63	H17A	C17	H17B	109.4

Number	Atom1	Atom2	Atom3	Angle/ °
64	H17A	C17	H17C	109.5
65	H17B	C17	H17C	109.5
66	F2	B1	F1	109.5(1)
67	F2	B1	N3	112.4(1)
68	F2	B1	N1	108.8(1)
69	F1	B1	N3	110.8(1)
70	F1	B1	N1	109.4(1)
71	N3	B1	N1	105.8(1)
72	H3A	O3	H3B	106(2)
73	H6A	O6	H6B	110(2)
74	H4A	O4	H4B	103(3)
75	H5A	O5	H5B	111(2)

Table S4. Torsion angles of **2a**

Number	Atom1	Atom2	Atom3	Atom4	Torsion/ °
1	C16	O2	C15	O1	0.4(2)
2	C16	O2	C15	C7	-179.5(1)
3	C15	O2	C16	H16A	53.8
4	C15	O2	C16	H16B	-66.2
5	C15	O2	C16	C17	173.8(1)
6	C6	N3	C14	C8	-1.9(1)
7	C6	N3	C14	C13	177.8(1)
8	B1	N3	C14	C8	166.2(1)
9	B1	N3	C14	C13	-14.1(2)
10	C14	N3	C6	N2	-177.5(1)
11	C14	N3	C6	C7	2.3(1)
12	B1	N3	C6	N2	13.8(2)
13	B1	N3	C6	C7	-166.4(1)
14	C14	N3	B1	F2	56.6(2)
15	C14	N3	B1	F1	-66.3(2)
16	C14	N3	B1	N1	175.1(1)
17	C6	N3	B1	F2	-136.8(1)
18	C6	N3	B1	F1	100.3(1)
19	C6	N3	B1	N1	-18.2(2)
20	C1	N1	C5	N2	-178.9(1)
21	C1	N1	C5	C4	0.7(2)
22	B1	N1	C5	N2	0.5(2)
23	B1	N1	C5	C4	-179.8(1)
24	C5	N1	C1	H1	179.5
25	C5	N1	C1	C2	-0.5(2)
26	B1	N1	C1	H1	-0.0
27	B1	N1	C1	C2	180.0(1)
28	C5	N1	B1	F2	132.5(1)
29	C5	N1	B1	F1	-107.9(1)
30	C5	N1	B1	N3	11.6(2)
31	C1	N1	B1	F2	-48.0(2)

Number	Atom1	Atom2	Atom3	Atom4	Torsion/ °
32	C1	N1	B1	F1	71.6(1)
33	C1	N1	B1	N3	-169.0(1)
34	C5	N2	C6	N3	0.9(2)
35	C5	N2	C6	C7	-178.8(1)
36	C6	N2	C5	N1	-8.0(2)
37	C6	N2	C5	C4	172.3(1)
38	C6	C7	C8	C14	0.6(1)
39	C6	C7	C8	C9	-178.2(1)
40	C15	C7	C8	C14	-178.8(1)
41	C15	C7	C8	C9	2.5(2)
42	C8	C7	C6	N3	-1.7(1)
43	C8	C7	C6	N2	178.0(1)
44	C15	C7	C6	N3	177.6(1)
45	C15	C7	C6	N2	-2.6(2)
46	C8	C7	C15	O2	15.1(2)
47	C8	C7	C15	O1	-164.9(1)
48	C6	C7	C15	O2	-164.1(1)
49	C6	C7	C15	O1	15.9(2)
50	N3	C14	C8	C7	0.8(1)
51	N3	C14	C8	C9	179.6(1)
52	C13	C14	C8	C7	-178.9(1)
53	C13	C14	C8	C9	-0.1(2)
54	N3	C14	C13	H13	0.7
55	N3	C14	C13	C12	-179.3(1)
56	C8	C14	C13	H13	-179.7
57	C8	C14	C13	C12	0.3(2)
58	C7	C8	C9	H9	-2.0
59	C7	C8	C9	C10	178.0(1)
60	C14	C8	C9	H9	179.5
61	C14	C8	C9	C10	-0.5(2)
62	N1	C5	C4	H4	179.5
63	N1	C5	C4	C3	-0.5(2)

Number	Atom1	Atom2	Atom3	Atom4	Torsion/ º
64	N2	C5	C4	H4	-0.8
65	N2	C5	C4	C3	179.2(1)
66	C14	C13	C12	H12	179.8
67	C14	C13	C12	C11	-0.2(3)
68	H13	C13	C12	H12	-0.2
69	H13	C13	C12	C11	179.8
70	C8	C9	C10	H10	-179.2
71	C8	C9	C10	C11	0.8(3)
72	H9	C9	C10	H10	0.8
73	H9	C9	C10	C11	-179.2
74	N1	C1	C2	H2	-179.9
75	N1	C1	C2	C3	0.1(2)
76	H1	C1	C2	H2	0.1
77	H1	C1	C2	C3	-179.9
78	C9	C10	C11	C12	-0.6(3)
79	C9	C10	C11	H11	179.4
80	H10	C10	C11	C12	179.4
81	H10	C10	C11	H11	-0.6
82	C5	C4	C3	C2	0.0(2)
83	C5	C4	C3	H3	-180.0
84	H4	C4	C3	C2	-180.0
85	H4	C4	C3	H3	0.0
86	O2	C16	C17	H17A	-57.8
87	O2	C16	C17	H17B	-177.8
88	O2	C16	C17	H17C	62.2
89	H16A	C16	C17	H17A	62.2
90	H16A	C16	C17	H17B	-57.8
91	H16A	C16	C17	H17C	-177.8
92	H16B	C16	C17	H17A	-177.8
93	H16B	C16	C17	H17B	62.2
94	H16B	C16	C17	H17C	-57.8
95	C13	C12	C11	C10	0.2(3)

Number	Atom1	Atom2	Atom3	Atom4	Torsion/ º
96	C13	C12	C11	H11	-179.7
97	H12	C12	C11	C10	-179.8
98	H12	C12	C11	H11	0.3
99	C1	C2	C3	C4	0.2(2)
100	C1	C2	C3	H3	-179.8
101	H2	C2	C3	C4	-179.8
102	H2	C2	C3	H3	0.2

Table S5. Bond lengths of **2b**

Number	Atom1	Atom2	Length/ nm
1	F1	B1	1.385(2)
2	F2	B1	1.376(2)
3	O2	C15	1.335(2)
4	O2	C16	1.453(2)
5	O1	C15	1.215(2)
6	O4	N4	1.220(2)
7	N3	C6	1.378(2)
8	N3	C14	1.383(2)
9	N3	B1	1.535(2)
10	N2	C6	1.335(2)
11	N2	C5	1.336(2)
12	N1	C5	1.366(2)
13	N1	C1	1.366(2)
14	N1	B1	1.576(3)
15	O3	N4	1.229(2)
16	N4	C4	1.473(2)
17	C6	C7	1.423(2)
18	C7	C15	1.473(2)
19	C7	C8	1.401(2)
20	C5	C4	1.423(3)
21	C14	C8	1.454(2)
22	C14	C13	1.380(2)
23	C8	C9	1.415(3)
24	C4	C3	1.356(3)
25	C9	H9	0.950
26	C9	C10	1.372(3)
27	C1	H1	0.950
28	C1	C2	1.361(3)
29	C13	H13	0.950
30	C13	C12	1.404(3)
31	C16	H16A	0.990
32	C16	H16B	0.990
33	C16	C17	1.499(3)
34	C12	H12	0.950
35	C12	C11	1.375(3)
36	C10	H10	0.950
37	C10	C11	1.403(3)
38	C3	H3	0.950
39	C3	C2	1.401(3)
40	C2	H2	0.950
41	C11	H11	0.950
42	C17	H17A	0.980
43	C17	H17B	0.980
44	C17	H17C	0.980

Table S6. Bond angles of **2b**

Number	Atom1	Atom2	Atom3	Angle/ °
1	C15	O2	C16	116.5(1)
2	C6	N3	C14	109.1(1)
3	C6	N3	B1	123.5(1)
4	C14	N3	B1	126.9(1)
5	C6	N2	C5	118.7(1)
6	C5	N1	C1	121.0(2)
7	C5	N1	B1	122.1(1)
8	C1	N1	B1	116.9(1)
9	O4	N4	O3	124.3(2)
10	O4	N4	C4	118.2(2)
11	O3	N4	C4	117.5(2)
12	N3	C6	N2	124.0(1)
13	N3	C6	C7	109.1(1)
14	N2	C6	C7	126.9(2)
15	C6	C7	C15	126.9(2)
16	C6	C7	C8	107.3(1)
17	C15	C7	C8	125.8(2)
18	N2	C5	N1	125.0(2)
19	N2	C5	C4	119.0(2)
20	N1	C5	C4	116.0(2)
21	N3	C14	C8	107.6(1)
22	N3	C14	C13	122.3(2)
23	C8	C14	C13	130.1(2)
24	O2	C15	O1	123.6(2)
25	O2	C15	C7	111.3(1)
26	O1	C15	C7	125.1(2)
27	C7	C8	C14	107.0(1)
28	C7	C8	C9	126.7(2)
29	C14	C8	C9	126.3(2)
30	N4	C4	C5	116.9(2)

Number	Atom1	Atom2	Atom3	Angle/ °
31	N4	C4	C3	119.8(2)
32	C5	C4	C3	123.4(2)
33	C8	C9	H9	115.8
34	C8	C9	C10	128.3(2)
35	H9	C9	C10	115.8
36	N1	C1	H1	118.6
37	N1	C1	C2	122.7(2)
38	H1	C1	C2	118.7
39	C14	C13	H13	116.5
40	C14	C13	C12	127.1(2)
41	H13	C13	C12	116.5
42	O2	C16	H16A	110.3
43	O2	C16	H16B	110.3
44	O2	C16	C17	107.0(1)
45	H16A	C16	H16B	108.5
46	H16A	C16	C17	110.3
47	H16B	C16	C17	110.4
48	C13	C12	H12	115.3
49	C13	C12	C11	129.3(2)
50	H12	C12	C11	115.4
51	C9	C10	H10	115.2
52	C9	C10	C11	129.5(2)
53	H10	C10	C11	115.2
54	C4	C3	H3	120.8
55	C4	C3	C2	118.3(2)
56	H3	C3	C2	120.8
57	C1	C2	C3	118.6(2)
58	C1	C2	H2	120.7
59	C3	C2	H2	120.7
60	C12	C11	C10	129.2(2)
61	C12	C11	H11	115.4
62	C10	C11	H11	115.4

Number	Atom1	Atom2	Atom3	Angle/ °
63	C16	C17	H17A	109.5
64	C16	C17	H17B	109.5
65	C16	C17	H17C	109.5
66	H17A	C17	H17B	109.5
67	H17A	C17	H17C	109.5
68	H17B	C17	H17C	109.5
69	F1	B1	F2	110.3(1)
70	F1	B1	N3	110.9(1)
71	F1	B1	N1	108.1(1)
72	F2	B1	N3	112.5(1)
73	F2	B1	N1	108.8(1)
74	N3	B1	N1	105.9(1)

Table S7. Torsion angles of **2b**

Number	Atom1	Atom2	Atom3	Atom4	Torsion/ °
1	C16	O2	C15	O1	0.6(2)
2	C16	O2	C15	C7	-178.9(1)
3	C15	O2	C16	H16A	47.1
4	C15	O2	C16	H16B	-72.8
5	C15	O2	C16	C17	167.1(1)
6	C14	N3	C6	N2	178.4(2)
7	C14	N3	C6	C7	0.1(2)
8	B1	N3	C6	N2	6.1(3)
9	B1	N3	C6	C7	-172.2(1)
10	C6	N3	C14	C8	-0.4(2)
11	C6	N3	C14	C13	179.9(2)
12	B1	N3	C14	C8	171.6(2)
13	B1	N3	C14	C13	-8.1(3)
14	C6	N3	B1	F1	107.2(2)
15	C6	N3	B1	F2	-128.6(2)
16	C6	N3	B1	N1	-9.8(2)
17	C14	N3	B1	F1	-63.7(2)
18	C14	N3	B1	F2	60.5(2)
19	C14	N3	B1	N1	179.2(1)
20	C5	N2	C6	N3	0.6(2)
21	C5	N2	C6	C7	178.7(2)
22	C6	N2	C5	N1	-1.6(3)
23	C6	N2	C5	C4	176.8(2)
24	C1	N1	C5	N2	179.8(2)
25	C1	N1	C5	C4	1.3(2)
26	B1	N1	C5	N2	-3.9(3)
27	B1	N1	C5	C4	177.6(2)
28	C5	N1	C1	H1	177.8
29	C5	N1	C1	C2	-2.1(3)
30	B1	N1	C1	H1	1.4

Number	Atom1	Atom2	Atom3	Atom4	Torsion/ °
31	B1	N1	C1	C2	-178.6(2)
32	C5	N1	B1	F1	-110.1(2)
33	C5	N1	B1	F2	130.0(2)
34	C5	N1	B1	N3	8.8(2)
35	C1	N1	B1	F1	66.2(2)
36	C1	N1	B1	F2	-53.6(2)
37	C1	N1	B1	N3	-174.8(1)
38	O4	N4	C4	C5	-106.6(2)
39	O4	N4	C4	C3	73.3(2)
40	O3	N4	C4	C5	73.9(2)
41	O3	N4	C4	C3	-106.2(2)
42	N3	C6	C7	C15	179.4(2)
43	N3	C6	C7	C8	0.2(2)
44	N2	C6	C7	C15	1.2(3)
45	N2	C6	C7	C8	-178.1(2)
46	C6	C7	C15	O2	18.4(2)
47	C6	C7	C15	O1	-161.1(2)
48	C8	C7	C15	O2	-162.5(2)
49	C8	C7	C15	O1	18.0(3)
50	C6	C7	C8	C14	-0.4(2)
51	C6	C7	C8	C9	-179.1(2)
52	C15	C7	C8	C14	-179.7(2)
53	C15	C7	C8	C9	1.6(3)
54	N2	C5	C4	N4	2.5(2)
55	N2	C5	C4	C3	-177.4(2)
56	N1	C5	C4	N4	-178.9(2)
57	N1	C5	C4	C3	1.2(3)
58	N3	C14	C8	C7	0.5(2)
59	N3	C14	C8	C9	179.2(2)
60	C13	C14	C8	C7	-179.8(2)
61	C13	C14	C8	C9	-1.0(3)
62	N3	C14	C13	H13	-3.1

Number	Atom1	Atom2	Atom3	Atom4	Torsion/ °
63	N3	C14	C13	C12	176.9(2)
64	C8	C14	C13	H13	177.2
65	C8	C14	C13	C12	-2.8(3)
66	C7	C8	C9	H9	1.6
67	C7	C8	C9	C10	-178.5(2)
68	C14	C8	C9	H9	-176.9
69	C14	C8	C9	C10	3.0(3)
70	N4	C4	C3	H3	-2.8
71	N4	C4	C3	C2	177.2(2)
72	C5	C4	C3	H3	177.1
73	C5	C4	C3	C2	-2.9(3)
74	C8	C9	C10	H10	179.9
75	C8	C9	C10	C11	-0.1(4)
76	H9	C9	C10	H10	-0.1
77	H9	C9	C10	C11	179.9
78	N1	C1	C2	C3	0.4(3)
79	N1	C1	C2	H2	-179.6
80	H1	C1	C2	C3	-179.5
81	H1	C1	C2	H2	0.5
82	C14	C13	C12	H12	-177.5
83	C14	C13	C12	C11	2.6(3)
84	H13	C13	C12	H12	2.5
85	H13	C13	C12	C11	-177.4
86	O2	C16	C17	H17A	65.2
87	O2	C16	C17	H17B	-54.8
88	O2	C16	C17	H17C	-174.8
89	H16A	C16	C17	H17A	-174.8
90	H16A	C16	C17	H17B	65.2
91	H16A	C16	C17	H17C	-54.8
92	H16B	C16	C17	H17A	-54.9
93	H16B	C16	C17	H17B	-174.9
94	H16B	C16	C17	H17C	65.1

Number	Atom1	Atom2	Atom3	Atom4	Torsion/ º
95	C13	C12	C11	C10	1.4(4)
96	C13	C12	C11	H11	-178.5
97	H12	C12	C11	C10	-178.5
98	H12	C12	C11	H11	1.6
99	C9	C10	C11	C12	-3.2(4)
100	C9	C10	C11	H11	176.8
101	H10	C10	C11	C12	176.8
102	H10	C10	C11	H11	-3.2
103	C4	C3	C2	C1	2.1(3)
104	C4	C3	C2	H2	-178.0
105	H3	C3	C2	C1	-177.9
106	H3	C3	C2	H2	2.0

Table S8. Bond lengths of **2c**

Number	Atom1	Atom2	Length/ nm
1	O2	C15	1.322(2)
2	O2	C16	1.449(2)
3	F2A	B1	1.340(9)
4	F1A	B1	1.45(1)
5	N1	C5	1.380(1)
6	N1	C1	1.354(1)
7	N1	B1	1.570(2)
8	N3	C14	1.377(1)
9	N3	C6	1.378(1)
10	N3	B1	1.536(2)
11	N2	C6	1.334(2)
12	N2	C5	1.327(2)
13	O4	N4	1.216(2)
14	O1A	C15	1.20(2)
15	N4	O3	1.226(1)
16	N4	C2	1.449(2)
17	C14	C8	1.456(2)
18	C14	C13	1.376(2)
19	C6	C7	1.420(2)
20	C5	C4	1.420(2)
21	C8	C7	1.391(2)
22	C8	C9	1.409(2)
23	C1	H1	0.930
24	C1	C2	1.363(2)
25	C2	C3	1.399(2)
26	C7	C15	1.471(2)
27	C13	H13	0.930
28	C13	C12	1.398(2)
29	C4	H4	0.930
30	C4	C3	1.351(2)
31	C9	H9	0.930
32	C9	C10	1.370(2)
33	C3	H3	0.930
34	C10	H10	0.930
35	C10	C11	1.396(2)
36	C11	H11	0.930
37	C11	C12	1.375(2)
38	C12	H12	0.930
39	C16	H16A	0.970
40	C16	H16B	0.970
41	C16	C17	1.477(2)
42	C17	H17A	0.960
43	C17	H17B	0.960
44	C17	H17C	0.960

Table S9. Bond angles of **2c**

Number	Atom1	Atom2	Atom3	Angle/ º
1	C15	O2	C16	117.4(1)
2	C5	N1	C1	120.4(1)
3	C5	N1	B1	122.5(1)
4	C1	N1	B1	117.0(1)
5	C14	N3	C6	109.0(1)
6	C14	N3	B1	128.2(1)
7	C6	N3	B1	122.8(1)
8	C6	N2	C5	119.4(1)
9	O4	N4	O3	123.7(1)
10	O4	N4	C2	118.8(1)
11	O3	N4	C2	117.6(1)
12	N3	C14	C8	107.7(1)
13	N3	C14	C13	122.6(1)
14	C8	C14	C13	129.7(1)
15	N3	C6	N2	124.6(1)
16	N3	C6	C7	108.9(1)
17	N2	C6	C7	126.6(1)
18	N1	C5	N2	123.8(1)
19	N1	C5	C4	118.2(1)
20	N2	C5	C4	118.0(1)
21	C14	C8	C7	106.8(1)
22	C14	C8	C9	126.1(1)
23	C7	C8	C9	127.1(1)
24	N1	C1	H1	119.5
25	N1	C1	C2	120.9(1)
26	H1	C1	C2	119.5
27	N4	C2	C1	119.0(1)
28	N4	C2	C3	120.2(1)
29	C1	C2	C3	120.9(1)
30	C6	C7	C8	107.6(1)

Number	Atom1	Atom2	Atom3	Angle/ °
31	C6	C7	C15	126.3(1)
32	C8	C7	C15	125.9(1)
33	O2	C15	O1A	125.7(7)
34	O2	C15	C7	112.3(1)
35	O1A	C15	C7	121.5(7)
36	C14	C13	H13	116.1
37	C14	C13	C12	127.9(1)
38	H13	C13	C12	116.1
39	C5	C4	H4	119.3
40	C5	C4	C3	121.4(1)
41	H4	C4	C3	119.3
42	C8	C9	H9	115.7
43	C8	C9	C10	128.5(1)
44	H9	C9	C10	115.7
45	C2	C3	C4	118.2(1)
46	C2	C3	H3	120.9
47	C4	C3	H3	120.9
48	C9	C10	H10	115.1
49	C9	C10	C11	129.9(1)
50	H10	C10	C11	115.1
51	C10	C11	H11	115.5
52	C10	C11	C12	129.0(1)
53	H11	C11	C12	115.5
54	C13	C12	C11	129.0(1)
55	C13	C12	H12	115.5
56	C11	C12	H12	115.5
57	O2	C16	H16A	110.0
58	O2	C16	H16B	110.0
59	O2	C16	C17	108.3(1)
60	H16A	C16	H16B	108.4
61	H16A	C16	C17	110.0
62	H16B	C16	C17	110.0

Number	Atom1	Atom2	Atom3	Angle/ °
63	C16	C17	H17A	109.5
64	C16	C17	H17B	109.5
65	C16	C17	H17C	109.5
66	H17A	C17	H17B	109.5
67	H17A	C17	H17C	109.5
68	H17B	C17	H17C	109.5
69	F2A	B1	F1A	111.5(6)
70	F2A	B1	N1	109.5(4)
71	F2A	B1	N3	115.4(4)
72	F1A	B1	N1	105.7(4)
73	F1A	B1	N3	107.9(4)
74	N1	B1	N3	106.2(1)

Table S10. Torsion angles of **2c**

Number	Atom1	Atom2	Atom3	Atom4	Torsion/ °
1	C16	O2	C15	O1A	2.0(8)
2	C16	O2	C15	C7	174.3(1)
3	C15	O2	C16	H16A	79.4
4	C15	O2	C16	H16B	-39.9
5	C15	O2	C16	C17	-160.2(1)
6	C1	N1	C5	N2	177.6(1)
7	C1	N1	C5	C4	-1.9(2)
8	B1	N1	C5	N2	-5.8(2)
9	B1	N1	C5	C4	174.7(1)
10	C5	N1	C1	H1	-179.2
11	C5	N1	C1	C2	0.8(2)
12	B1	N1	C1	H1	4.0
13	B1	N1	C1	C2	-176.0(1)
14	C5	N1	B1	F2A	134.7(4)
15	C5	N1	B1	F1A	-105.1(5)
16	C5	N1	B1	N3	9.4(2)
17	C1	N1	B1	F2A	-48.6(4)
18	C1	N1	B1	F1A	71.7(5)
19	C1	N1	B1	N3	-173.9(1)
20	C6	N3	C14	C8	1.4(1)
21	C6	N3	C14	C13	-178.0(1)
22	B1	N3	C14	C8	178.3(1)
23	B1	N3	C14	C13	-1.1(2)
24	C14	N3	C6	N2	179.7(1)
25	C14	N3	C6	C7	-1.3(1)
26	B1	N3	C6	N2	2.5(2)
27	B1	N3	C6	C7	-178.4(1)
28	C14	N3	B1	F2A	54.0(4)
29	C14	N3	B1	F1A	-71.4(5)
30	C14	N3	B1	N1	175.6(1)

Number	Atom1	Atom2	Atom3	Atom4	Torsion/ °
31	C6	N3	B1	F2A	-129.4(4)
32	C6	N3	B1	F1A	105.1(5)
33	C6	N3	B1	N1	-7.8(2)
34	C5	N2	C6	N3	2.8(2)
35	C5	N2	C6	C7	-176.0(1)
36	C6	N2	C5	N1	-1.1(2)
37	C6	N2	C5	C4	178.4(1)
38	O4	N4	C2	C1	-0.9(2)
39	O4	N4	C2	C3	177.7(1)
40	O3	N4	C2	C1	179.4(1)
41	O3	N4	C2	C3	-2.0(2)
42	N3	C14	C8	C7	-0.9(1)
43	N3	C14	C8	C9	-179.1(1)
44	C13	C14	C8	C7	178.4(1)
45	C13	C14	C8	C9	0.3(2)
46	N3	C14	C13	H13	0.9
47	N3	C14	C13	C12	-179.1(1)
48	C8	C14	C13	H13	-178.4
49	C8	C14	C13	C12	1.6(2)
50	N3	C6	C7	C8	0.7(1)
51	N3	C6	C7	C15	-176.2(1)
52	N2	C6	C7	C8	179.7(1)
53	N2	C6	C7	C15	2.8(2)
54	N1	C5	C4	H4	-178.6
55	N1	C5	C4	C3	1.4(2)
56	N2	C5	C4	H4	1.9
57	N2	C5	C4	C3	-178.1(1)
58	C14	C8	C7	C6	0.2(1)
59	C14	C8	C7	C15	177.1(1)
60	C9	C8	C7	C6	178.3(1)
61	C9	C8	C7	C15	-4.8(2)
62	C14	C8	C9	H9	178.3

Number	Atom1	Atom2	Atom3	Atom4	Torsion/ º
63	C14	C8	C9	C10	-1.7(2)
64	C7	C8	C9	H9	0.6
65	C7	C8	C9	C10	-179.4(1)
66	N1	C1	C2	N4	179.5(1)
67	N1	C1	C2	C3	0.9(2)
68	H1	C1	C2	N4	-0.5
69	H1	C1	C2	C3	-179.1
70	N4	C2	C3	C4	-180.0(1)
71	N4	C2	C3	H3	0.0
72	C1	C2	C3	C4	-1.4(2)
73	C1	C2	C3	H3	178.6
74	C6	C7	C15	O2	-28.8(2)
75	C6	C7	C15	O1A	143.9(8)
76	C8	C7	C15	O2	154.9(1)
77	C8	C7	C15	O1A	-32.5(8)
78	C14	C13	C12	C11	-0.9(3)
79	C14	C13	C12	H12	179.1
80	H13	C13	C12	C11	179.1
81	H13	C13	C12	H12	-0.9
82	C5	C4	C3	C2	0.2(2)
83	C5	C4	C3	H3	-179.8
84	H4	C4	C3	C2	-179.8
85	H4	C4	C3	H3	0.2
86	C8	C9	C10	H10	-179.8
87	C8	C9	C10	C11	0.2(3)
88	H9	C9	C10	H10	0.2
89	H9	C9	C10	C11	-179.8
90	C9	C10	C11	H11	-177.9
91	C9	C10	C11	C12	2.1(3)
92	H10	C10	C11	H11	2.1
93	H10	C10	C11	C12	-177.9
94	C10	C11	C12	C13	-1.6(3)

Number	Atom1	Atom2	Atom3	Atom4	Torsion/ °
95	C10	C11	C12	H12	178.4
96	H11	C11	C12	C13	178.4
97	H11	C11	C12	H12	-1.6
98	O2	C16	C17	H17A	-68.8
99	O2	C16	C17	H17B	171.2
100	O2	C16	C17	H17C	51.2
101	H16A	C16	C17	H17A	51.5
102	H16A	C16	C17	H17B	-68.5
103	H16A	C16	C17	H17C	171.5
104	H16B	C16	C17	H17A	170.8
105	H16B	C16	C17	H17B	50.9
106	H16B	C16	C17	H17C	-69.2

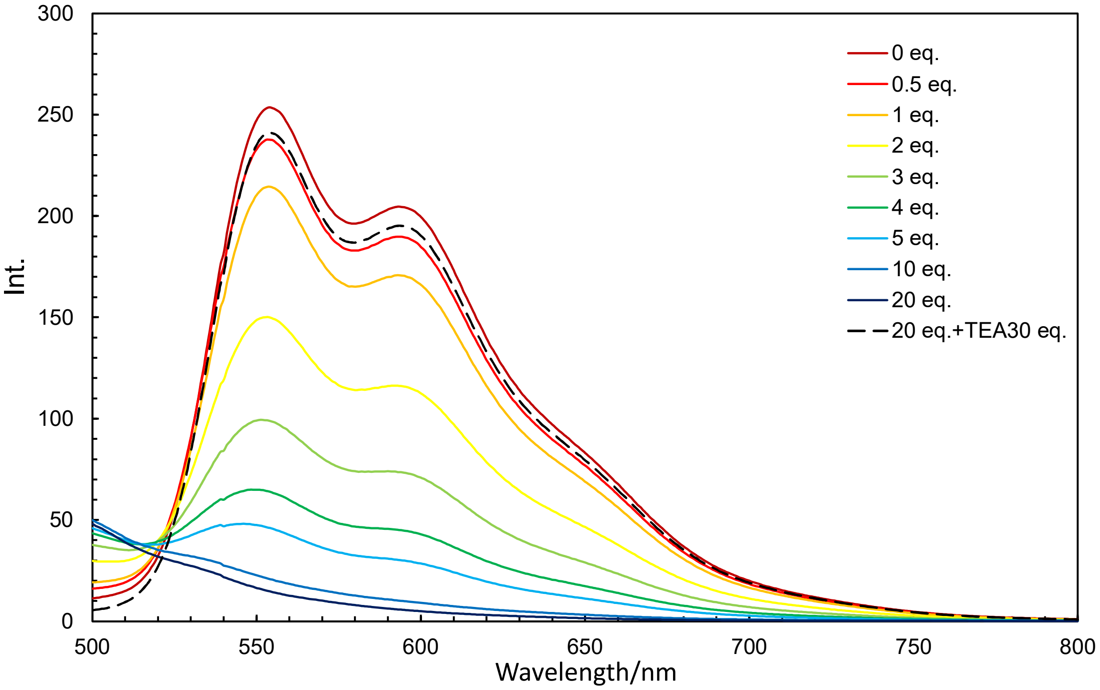

2a_485 nm

Fig. S2 Fluorescence spectra of **2a** (ex. 485 nm).

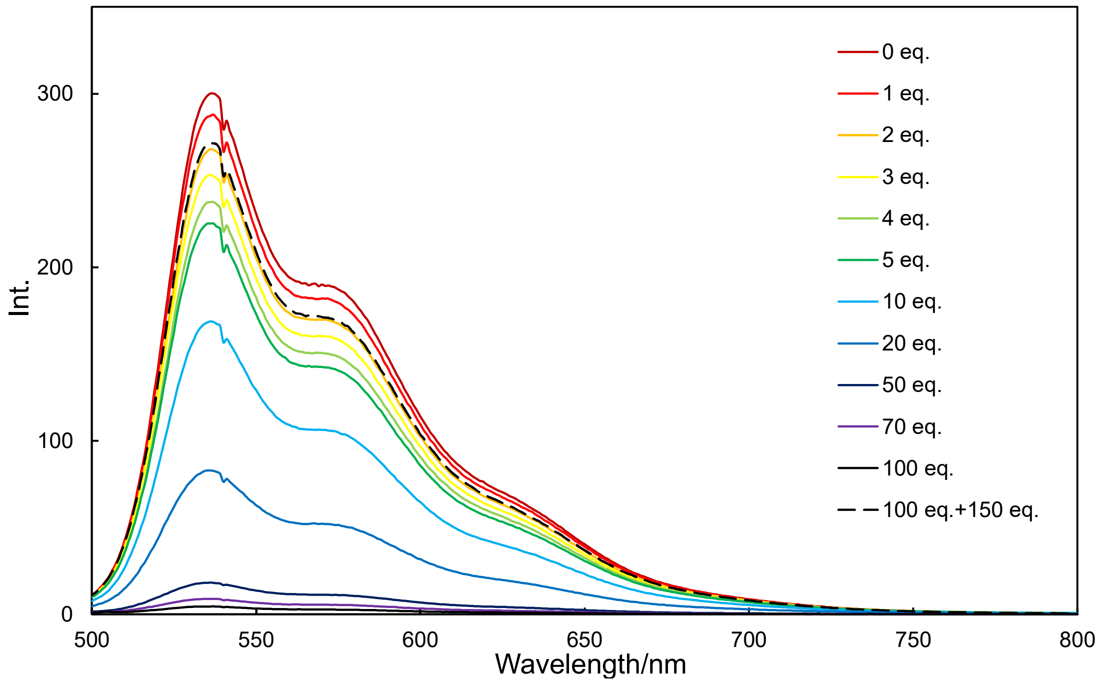

Fig. S3 Fluorescence spectra of **2b** (ex. 480 nm).

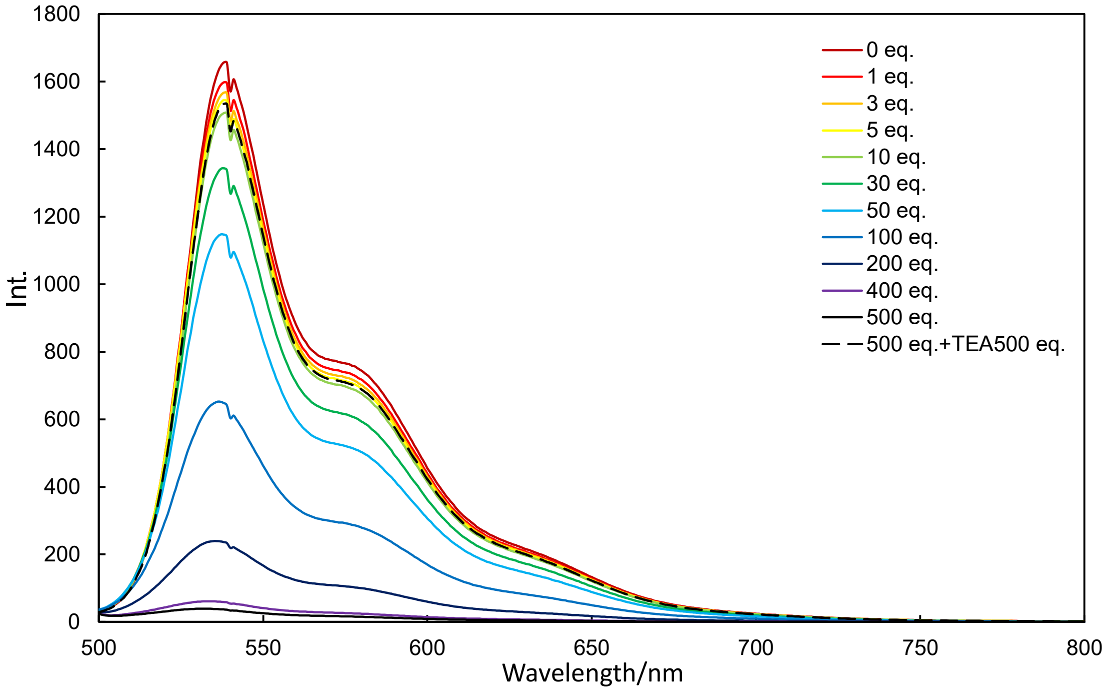

Fig. S4 Fluorescence spectra of **2c** (ex. 490 nm).

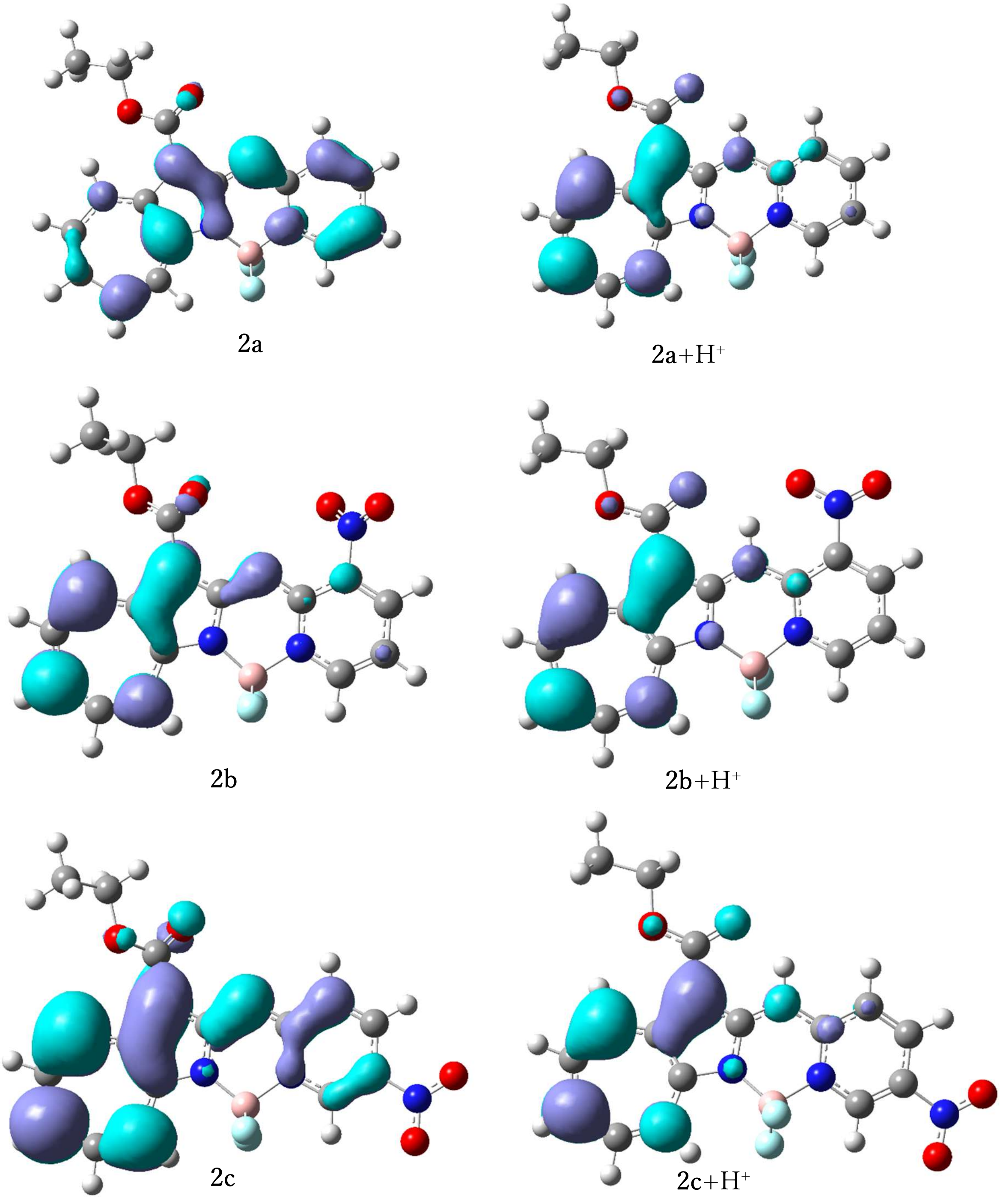

Fig. S5 Electron density difference plots for **2a** (upper left), **2a**+H^+ (upper right), **2b** (center left), **2b**+H^+ (center right), **2c** (lower left), and **2c**+H^+ (lower right). The light blue zones indicate electron density loss upon transition (donor) whereas the purple zones correspond to increase of electron density upon transition (acceptors).

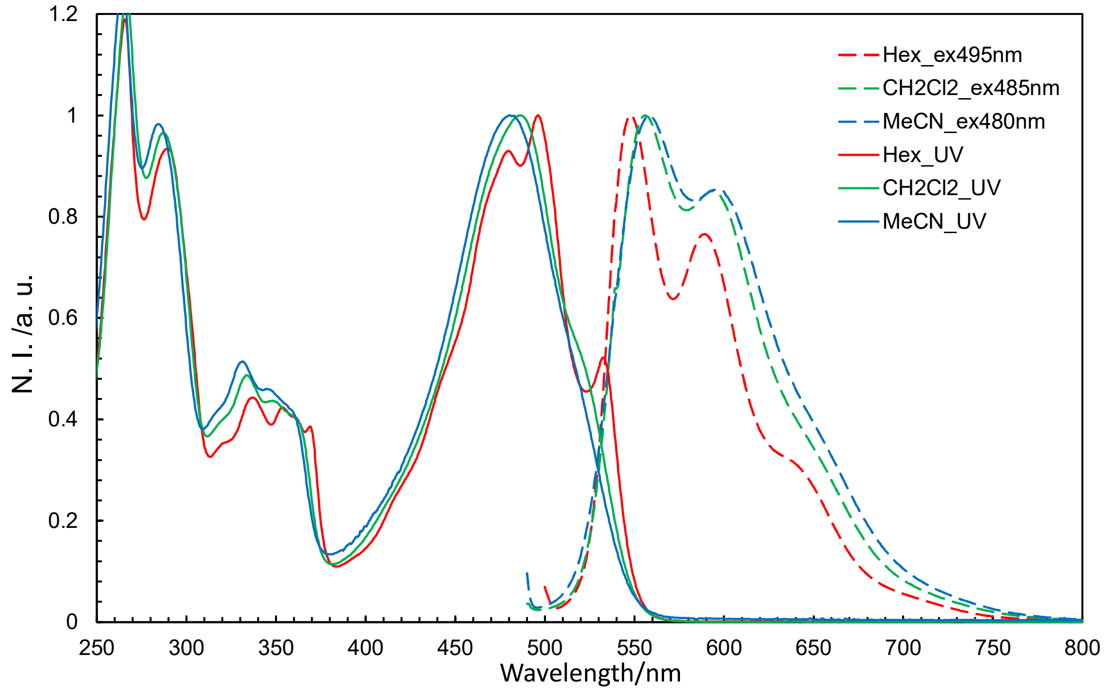

Fig. S6 Solvent dependence of **2a**.

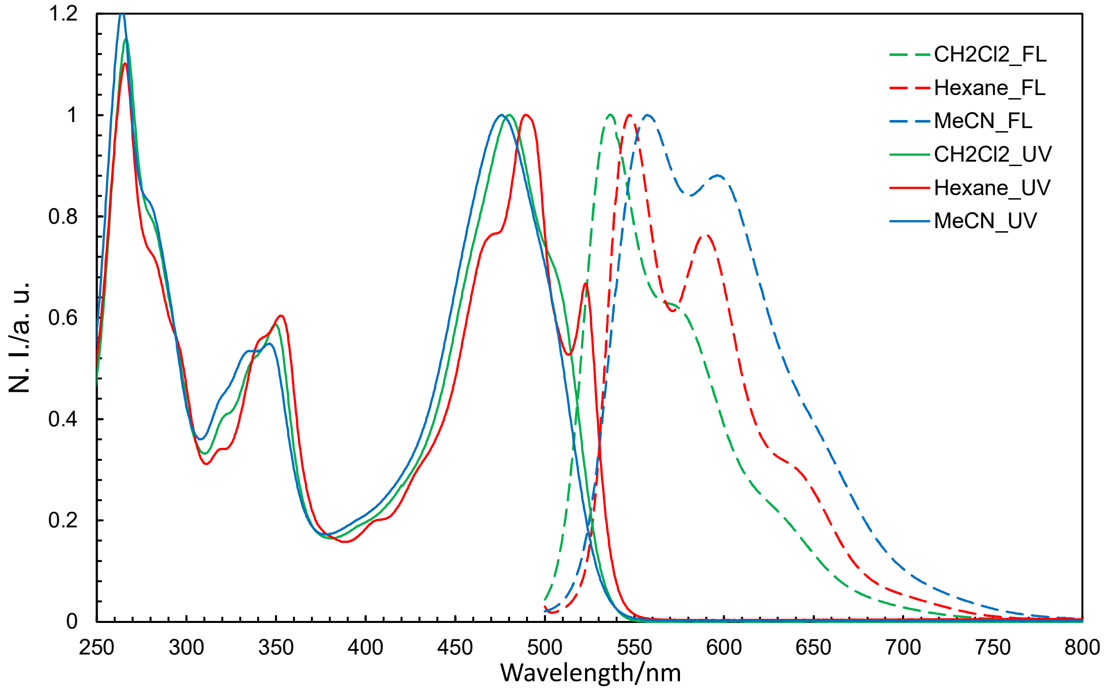

Fig. S7 Solvent dependence of **2b**.

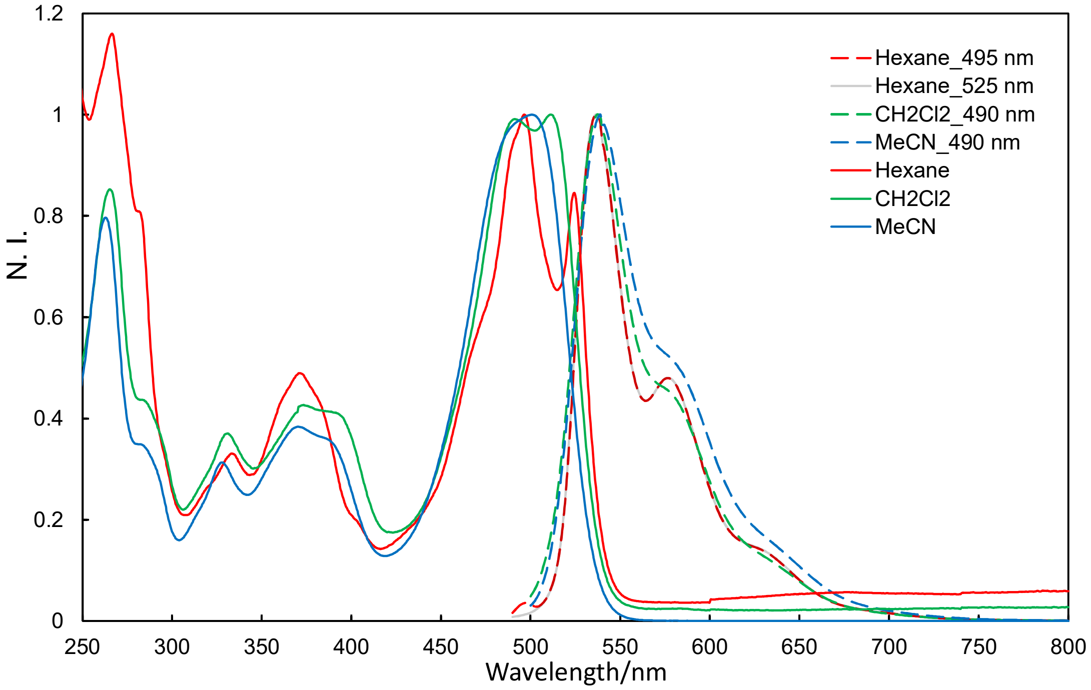

Fig. S8 Solvent dependence of **2c**.

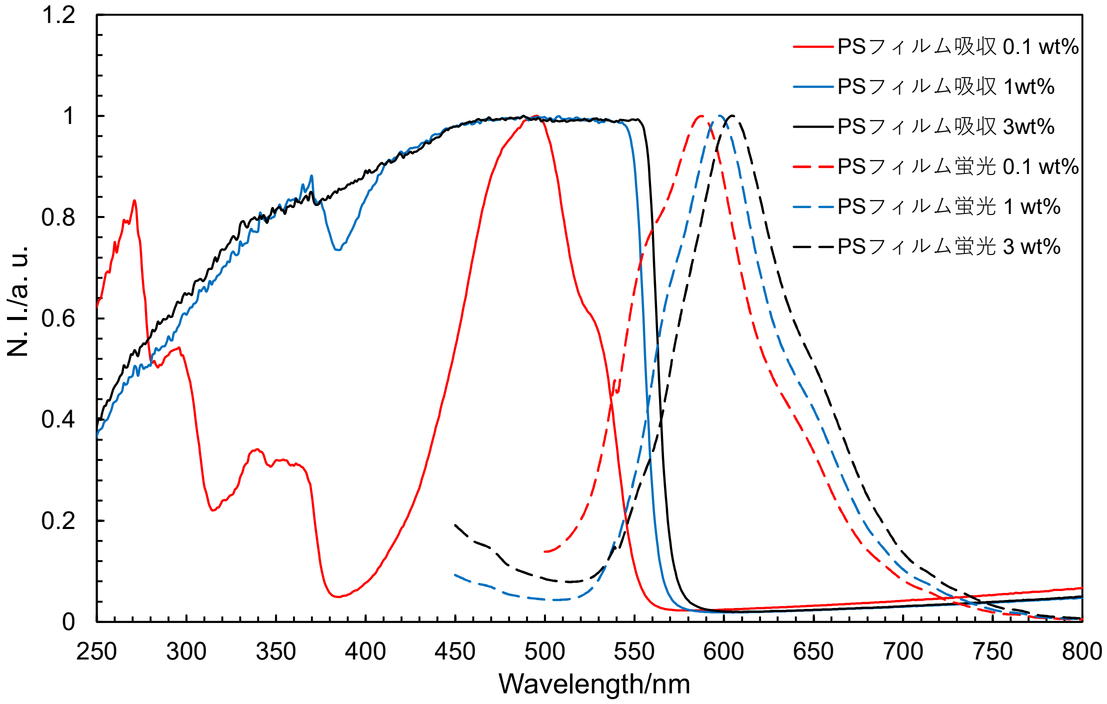

Fig. S9 UV–vis and fluorescence spectra of PS films added with **2a**.

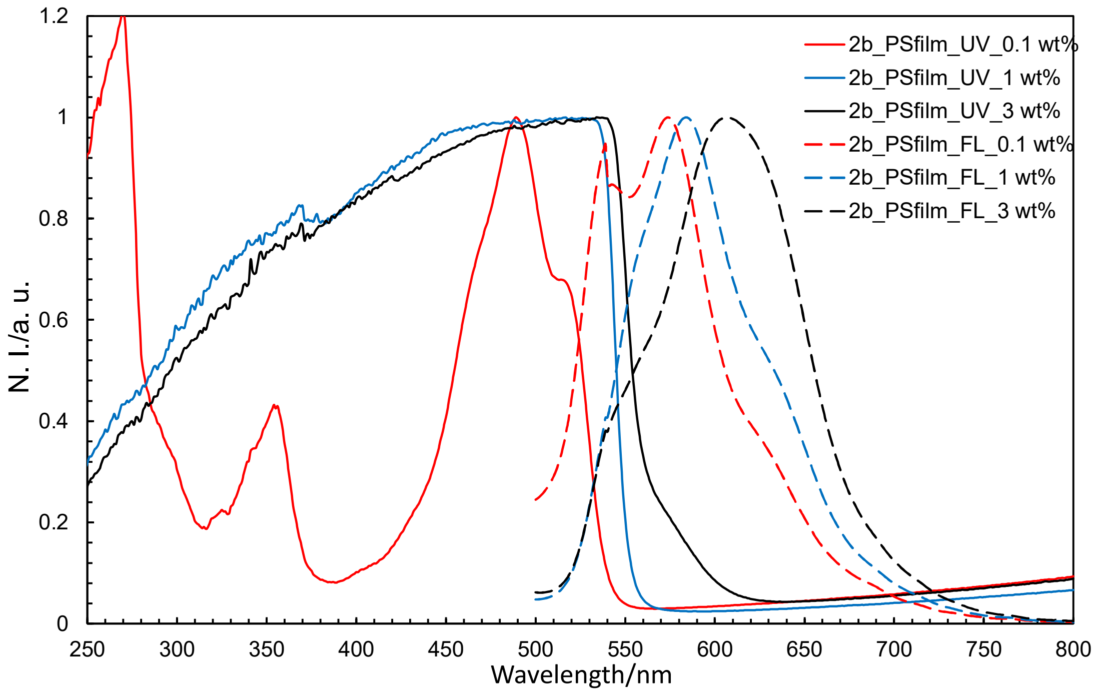

Fig. S10 UV–vis and fluorescence spectra of PS films added with **2b**.

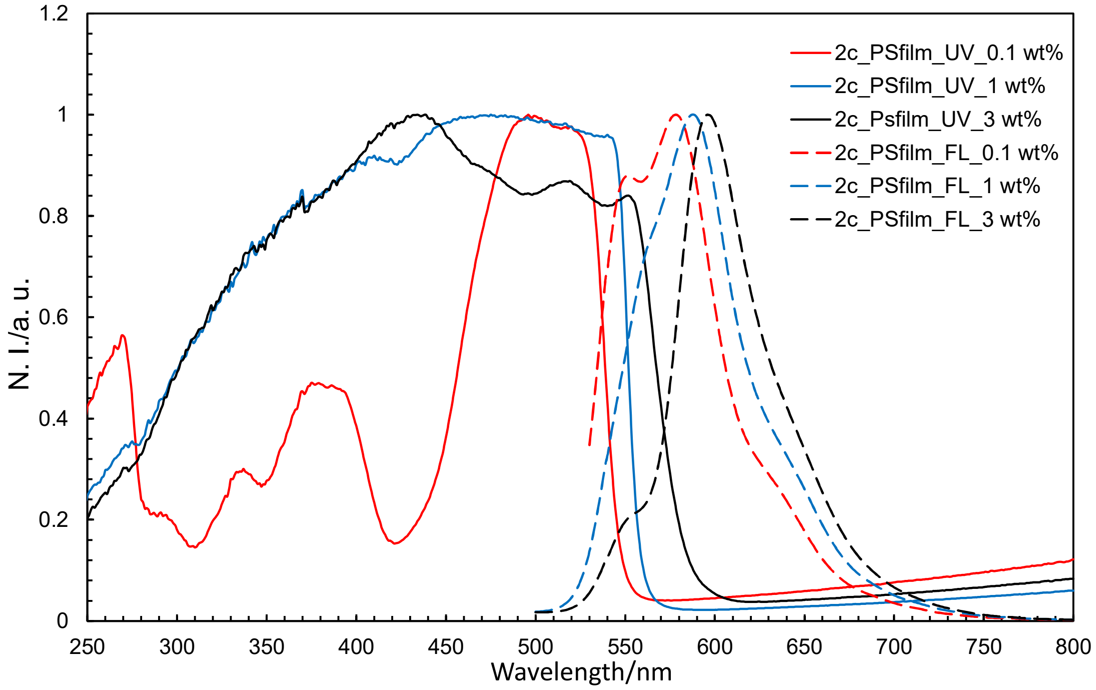

Fig. S11 UV–vis and fluorescence spectra of PS films added with **2c**.

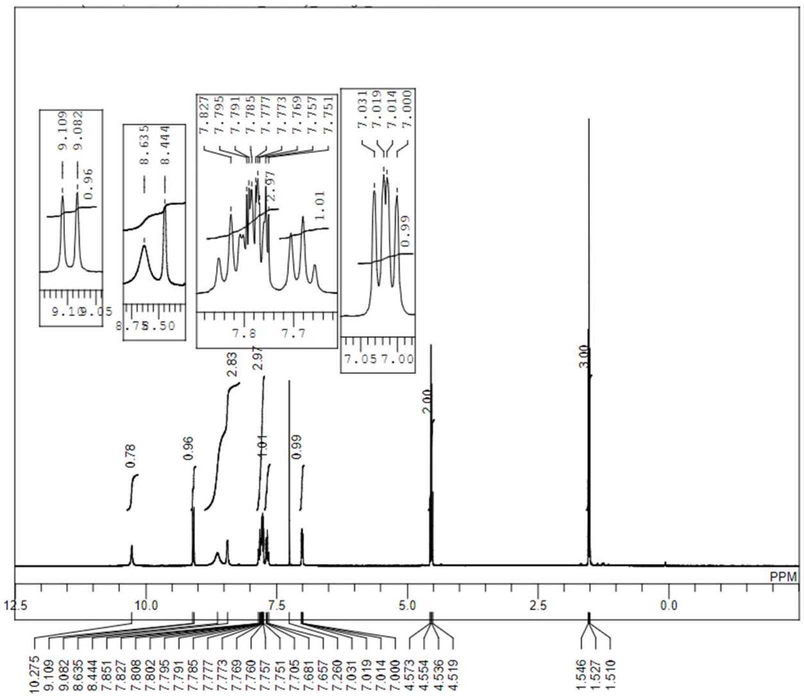

Fig. S12 ^{1}H NMR spectrum of **1a**.

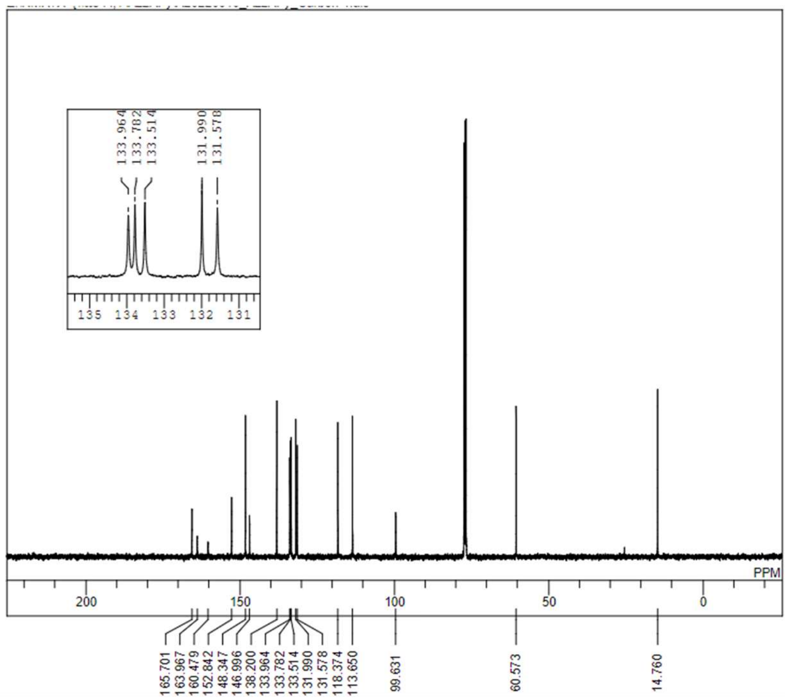

Fig. S13 ^{13}C NMR spectrum of **1a**.

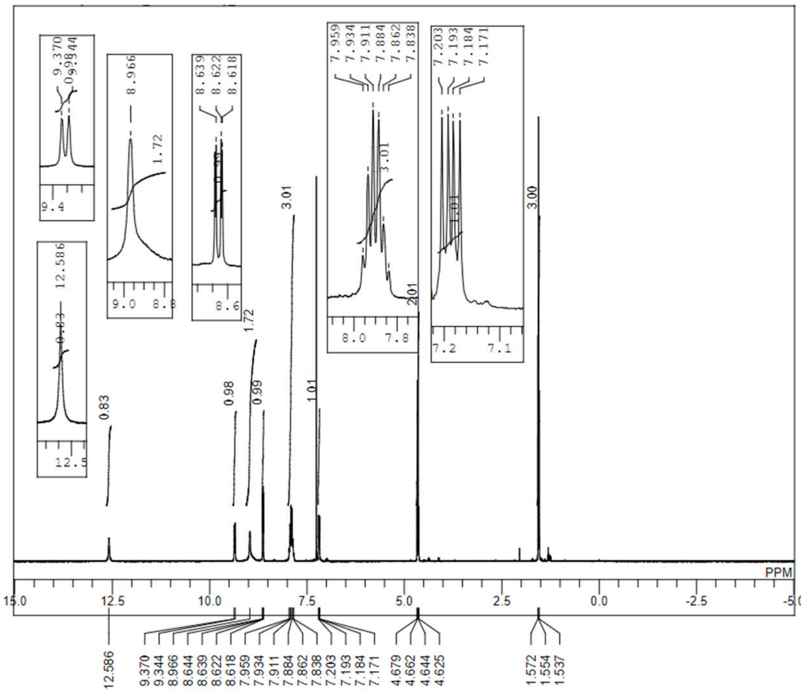

Fig. S14 ^{1}H NMR spectrum of **1b**.

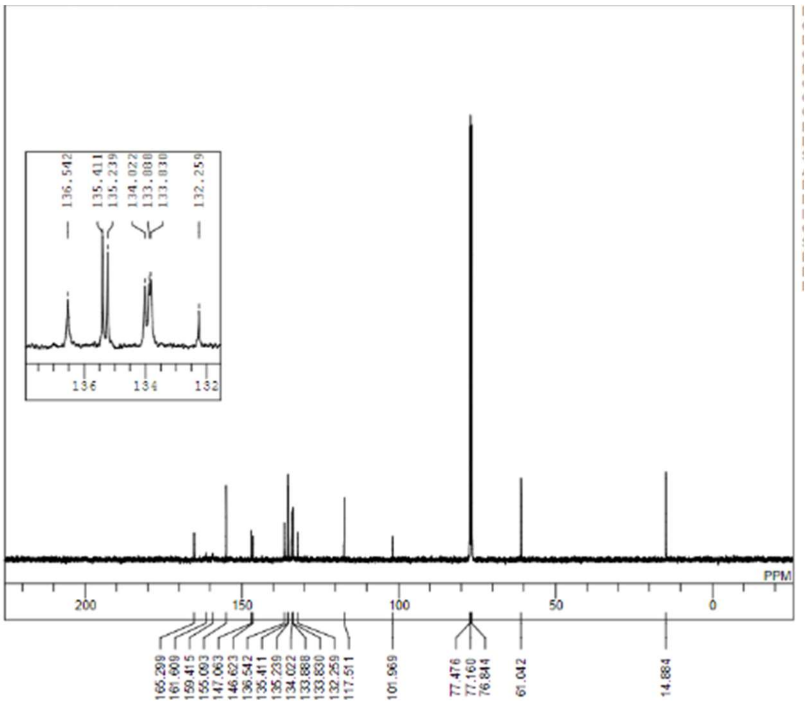

Fig. S15 ^{13}C NMR spectrum of **1b**.

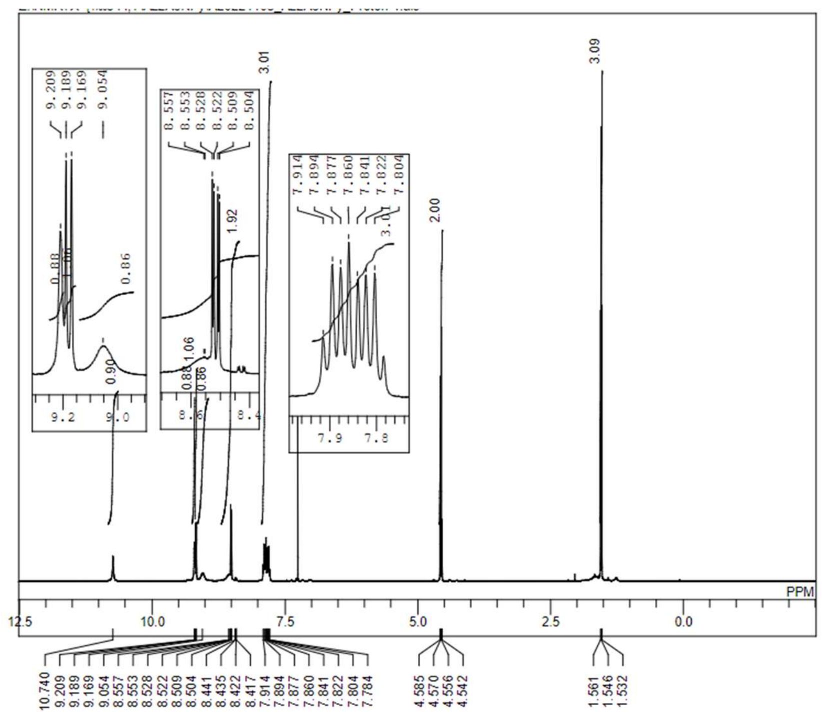

Fig. S16 ^{1}H NMR spectrum of **1c** at r.t.

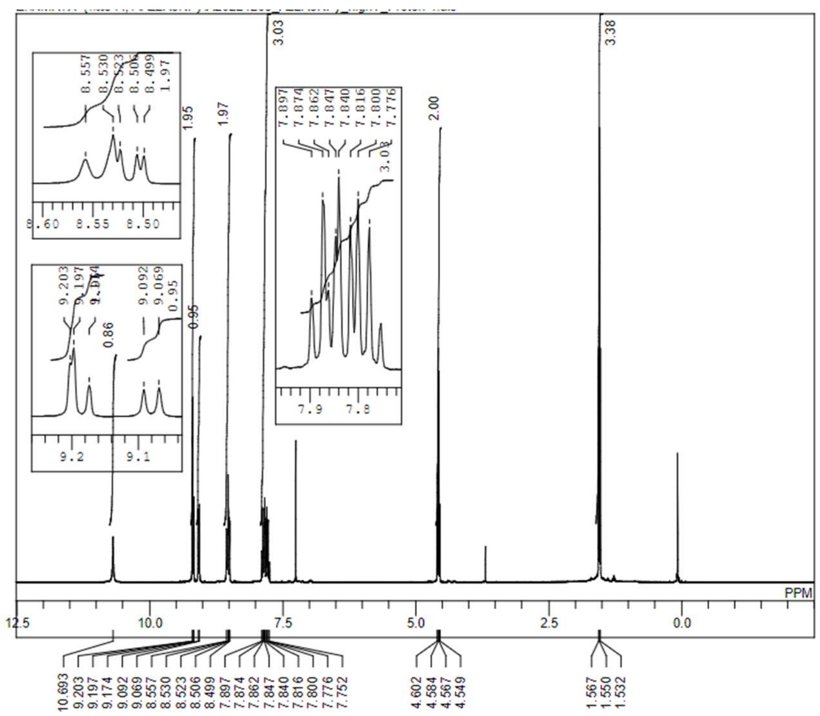

Fig. S17 ^{1}H NMR spectrum of **1c** at 55ºC.

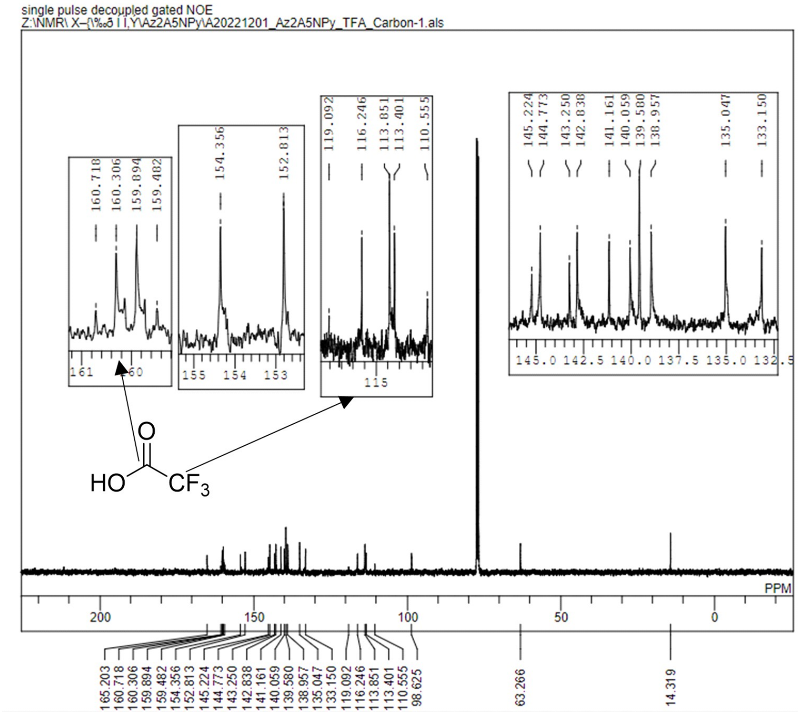

Fig. S18 ^{13}C NMR spectrum of **1c**+TFA.

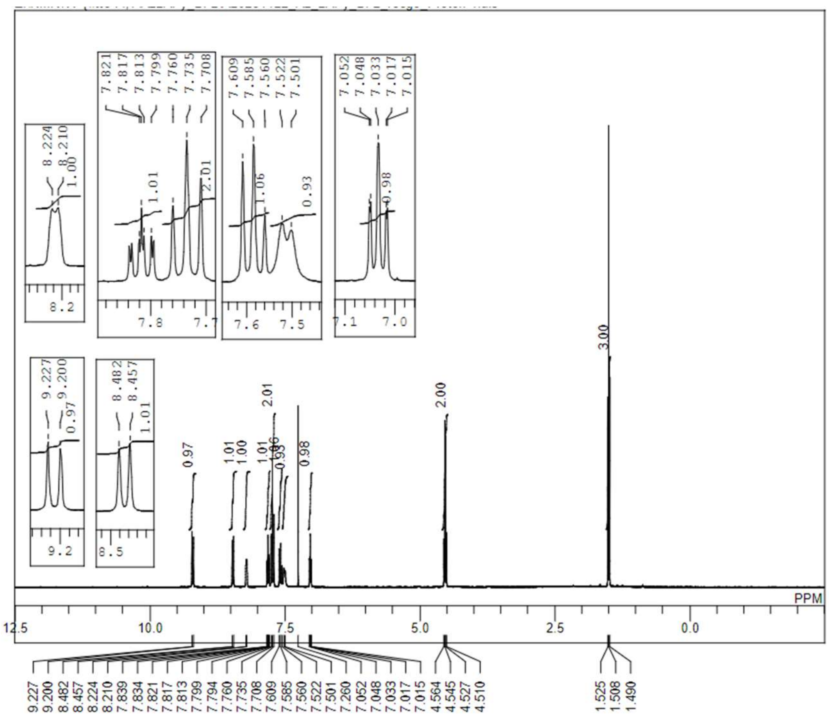

Fig. S19 ^{1}H NMR spectrum of **2a**.

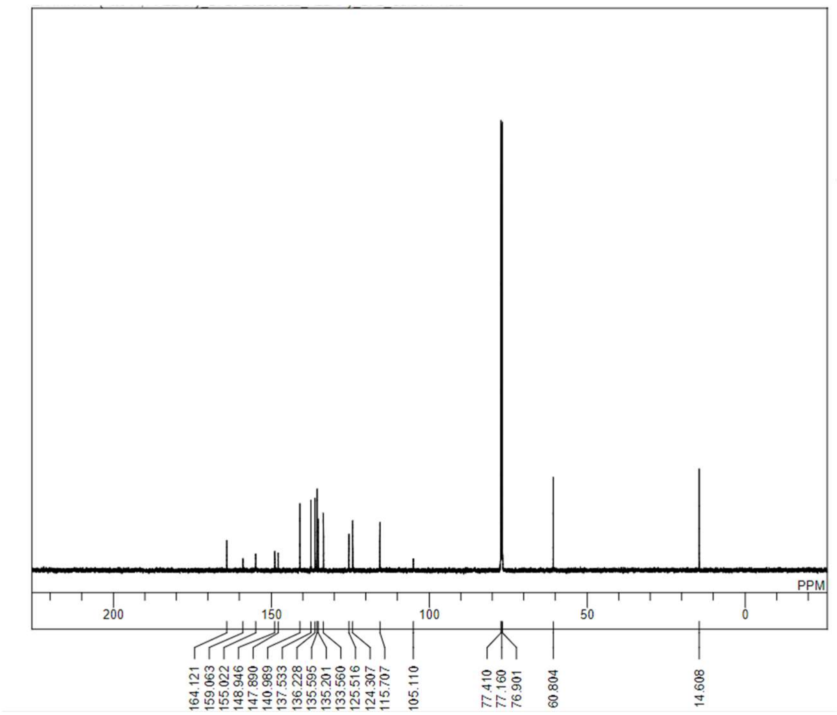

Fig. S20 ^{13}C NMR spectrum of **2a**.

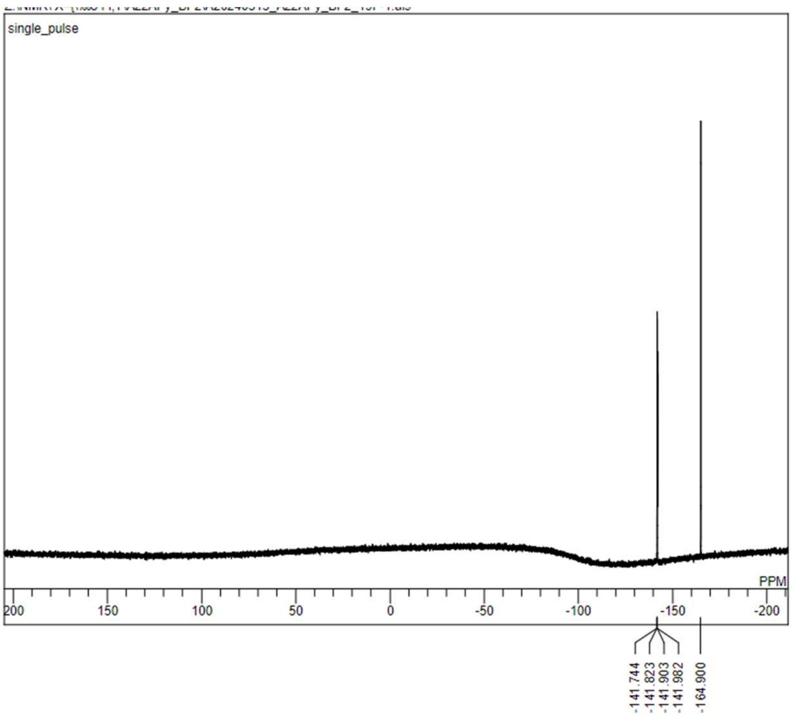

Fig. S21 ^{19}F NMR spectrum of **2a**.

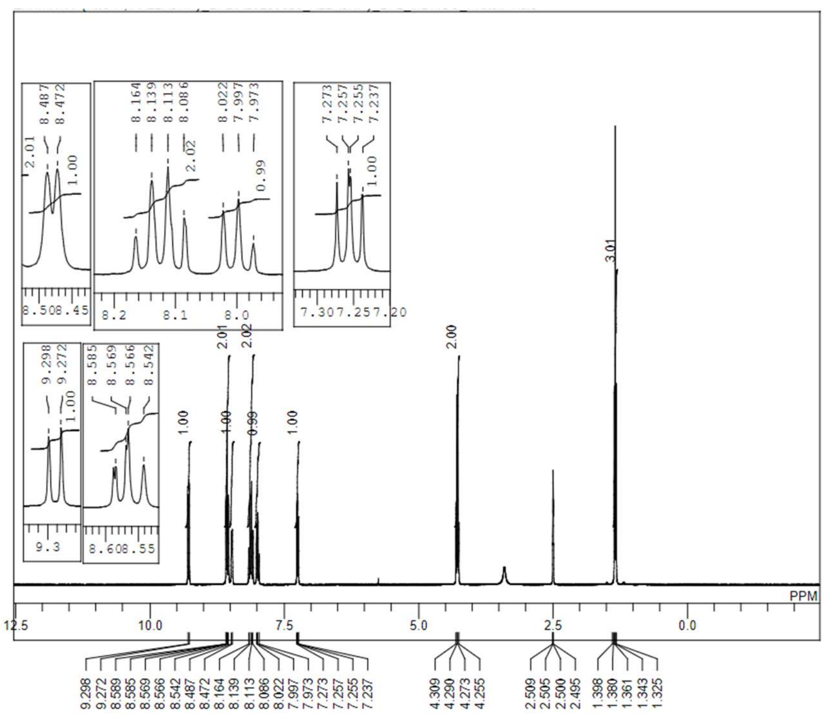

Fig. S22 ^{1}H NMR spectrum of **2b**.

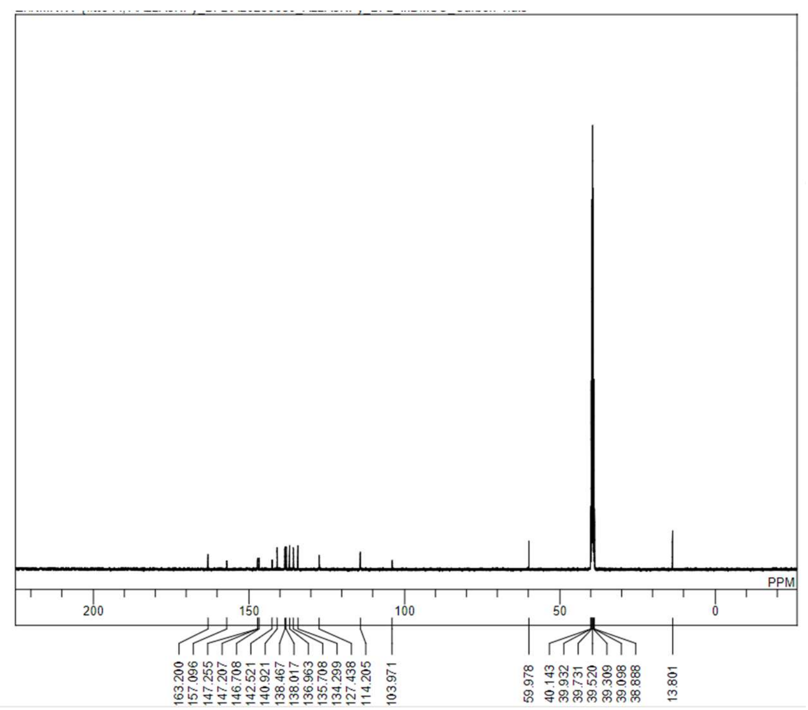

Fig. S23 ^{13}C NMR spectrum of **2b**.

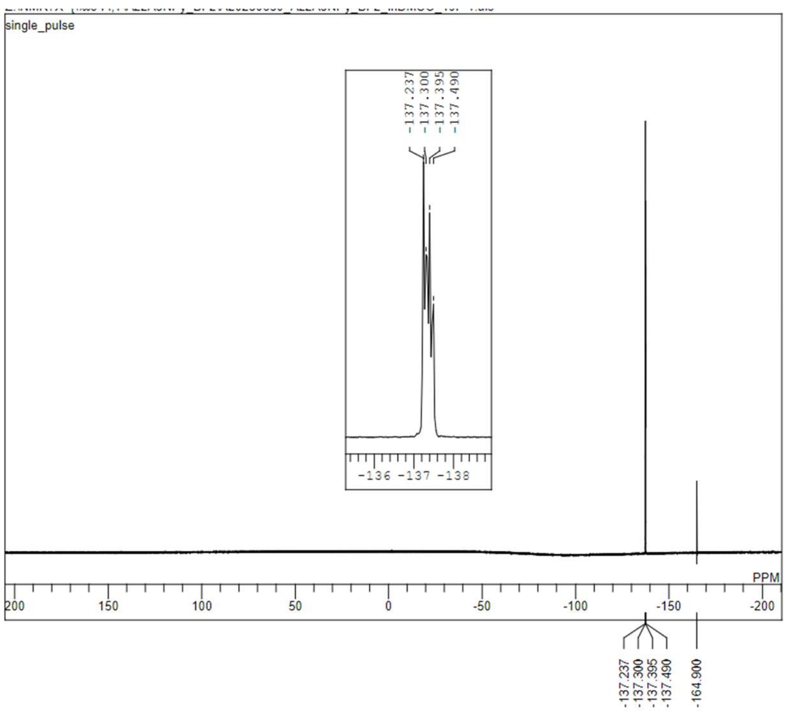

Fig. S24 ^{19}F NMR spectrum of **2b**.

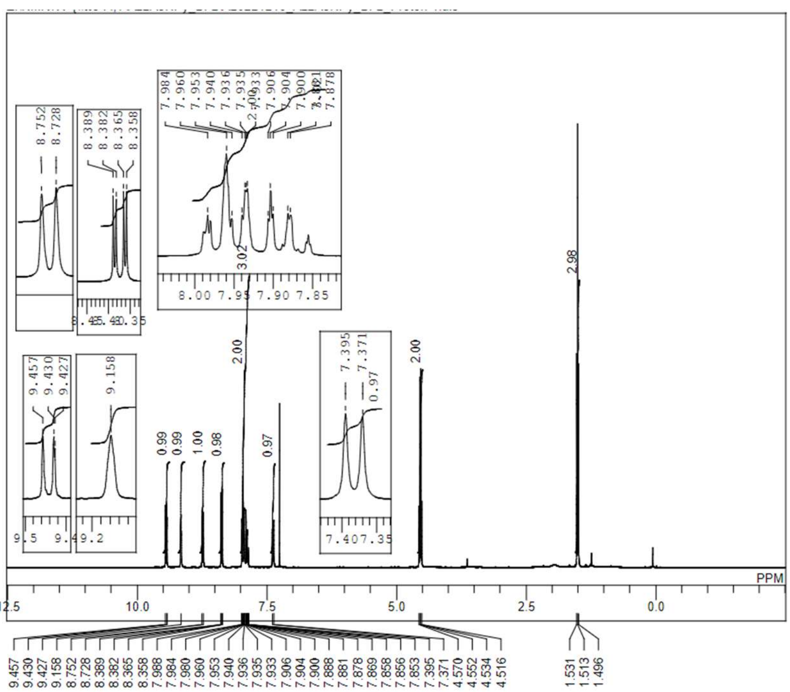

Fig. S25 ^{1}H NMR spectrum of **2c**.

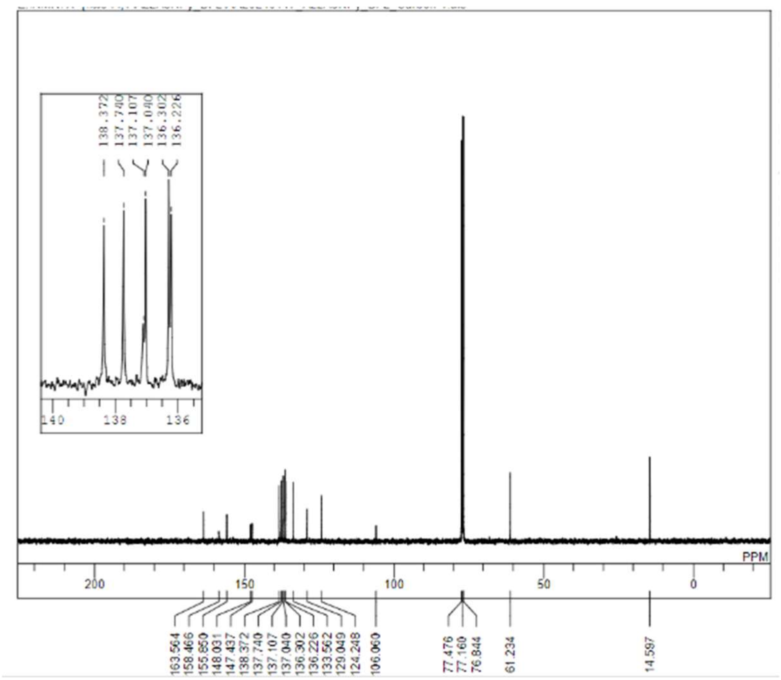

Fig. S26 ^{13}C NMR spectrum of **2c**.

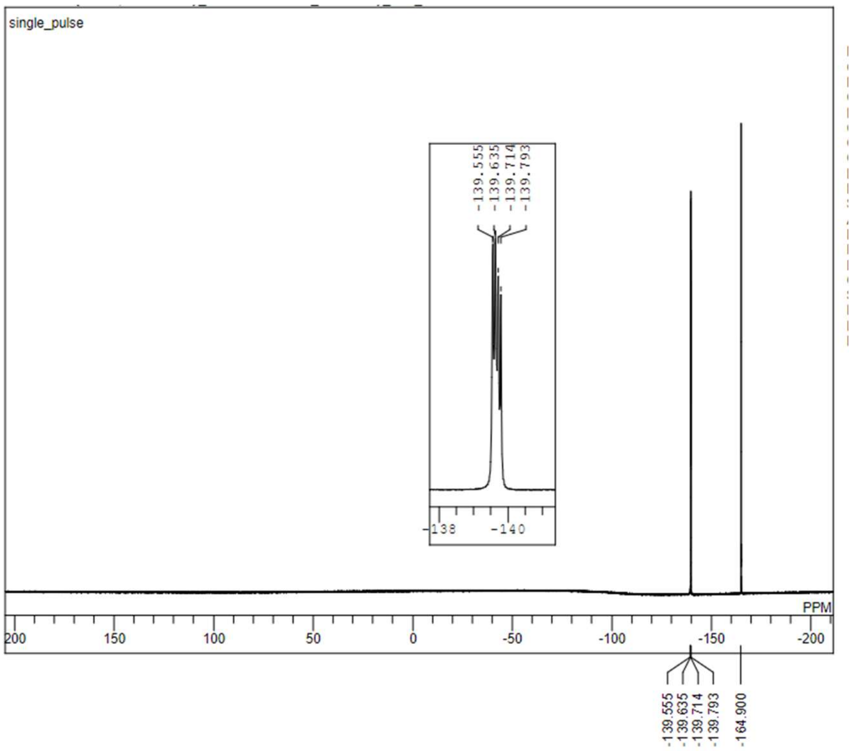

Fig. S27 ^{19}F NMR spectrum of **2c**.

DFT calculation

S_0 minimum energy structure of **2a** (B3LYP 6-31g+(d, p))

No imaginary frequency

Zero-point correction = 0.292888 (Hartree)

Thermal correction to Energy = 0.313644

Thermal correction to Enthalpy = 0.314588

Thermal correction to Gibbs Free Energy = 0.240383

Sum of electronic and zero-point Energies = -1195.454704

Sum of electronic and thermal Energies = -1195.433948

Sum of electronic and Enthalpies = -1195.433004

Sum of electronic and thermal Free Energies = -1195.507209

C	-2.70146	3.746294	-0.00026
C	-1.32706	3.931535	-1.7E-05
C	-0.30409	2.970238	0.000015
C	-3.414	2.530402	-0.00053
C	-0.42388	1.585942	-0.00017
C	-2.95401	1.224067	-0.00058
C	-1.62184	0.741128	-0.00042
H	-3.30697	4.649198	-0.00025
H	-0.985	4.963576	0.000166
H	0.710663	3.352785	0.000203
H	-4.49713	2.631819	-0.0007
H	-3.71307	0.452852	-0.00074
C	0.26428	-0.56391	-0.00039
C	-1.17562	-0.59631	-0.00062
C	2.424355	-1.38162	0.000037
C	3.285694	-2.51339	0.000142
N	2.999617	-0.13098	0.000217

C	4.65222	-2.35626	0.000397
H	2.808898	-3.48602	-1.2E-05
C	4.353814	0.017033	0.000461
C	5.209433	-1.05724	0.000557
H	4.705373	1.041957	0.000577
H	6.280658	-0.89595	0.00075
C	-1.94312	-1.85189	-0.00119
O	-1.46715	-2.97032	-0.0033
N	1.093589	-1.5901	-0.00026
O	-3.29635	-1.64527	0.000869
C	-4.11178	-2.84168	0.000347
C	-5.56621	-2.40813	0.002664
H	-3.86042	-3.43797	0.882609
H	-3.86255	-3.43577	-0.884
H	-6.21246	-3.29216	0.002438
H	-5.8008	-1.81527	0.892499
H	-5.80304	-1.81321	-0.8852
H	5.300292	-3.22769	0.000466
N	0.671044	0.763943	-0.00012
B	2.152048	1.195978	0.000251
F	2.464303	1.93904	-1.14362
F	2.463833	1.938669	1.144497

S_0 minimum energy structure of **2a**+H^+ (B3LYP 6-31g+(d, p))

No imaginary frequency

Zero-point correction = 0.307068 (Hartree)

Thermal correction to Energy = 0.327803

Thermal correction to Enthalpy = 0.328747

Thermal correction to Gibbs Free Energy = 0.256380

Sum of electronic and zero-point Energies = -1195.852060

Sum of electronic and thermal Energies = -1195.831324

Sum of electronic and Enthalpies = -1195.830380

Sum of electronic and thermal Free Energies = -1195.902747

C	-2.77406	3.786289	0.000015
C	-1.39708	3.994857	-0.00013
C	-0.36374	3.050102	-0.00019
C	-3.48415	2.573505	0.000087
C	-0.48344	1.665775	-0.00021
C	-3.00741	1.26846	0.000005
C	-1.67179	0.824911	-1.9E-05
H	-3.38527	4.685031	0.000072
H	-1.07384	5.032058	-0.00018
H	0.648113	3.442949	-0.00017
H	-4.56672	2.669692	0.000176
H	-3.75296	0.480914	-3.7E-05
C	0.191442	-0.446	-0.00011
C	-1.21289	-0.52174	-4.7E-05
C	2.434305	-1.36089	-4.7E-05
C	3.253565	-2.50056	-0.00012
N	2.970778	-0.11668	-5.3E-05
C	4.628053	-2.33879	-0.00015
H	2.796456	-3.48377	-0.00017
C	4.324788	0.033493	-6.2E-05
C	5.179366	-1.0463	-0.00012
H	4.675459	1.058272	-3.3E-05
H	6.250469	-0.88588	-0.00012
C	-1.89417	-1.82014	0.000063
O	-1.2623	-2.87724	0.000299
N	1.063874	-1.48551	0.000012
O	-3.22549	-1.75103	-4.3E-05
C	-3.95669	-3.02509	0.000099
C	-5.43573	-2.70249	-0.0002
H	-3.64986	-3.58458	0.887074

H	-3.64959	-3.58494	-0.88655
H	-6.00499	-3.63705	-0.00011
H	-5.71965	-2.13407	0.890329
H	-5.71936	-2.13439	-0.89102
H	5.273681	-3.21095	-0.00023
N	0.625462	0.837191	-0.0002
B	2.126196	1.252186	0.000149
F	2.441232	1.955725	-1.14553
F	2.44086	1.955112	1.146261
H	0.607624	-2.40305	0.000092

S_1 minimum energy structure of **2a** (B3LYP 6-31g+(d, p))

No imaginary frequency

Zero-point correction = 0.289587 (Hartree)

Thermal correction to Energy = 0.310770

Thermal correction to Enthalpy = 0.311715

Thermal correction to Gibbs Free Energy = 0.237786

Sum of electronic and zero-point Energies = -1195.371793

Sum of electronic and thermal Energies = -1195.350610

Sum of electronic and Enthalpies = -1195.349666

Sum of electronic and thermal Free Energies = -1195.423594

C	-2.80083	3.740021	-0.07731
C	-1.37384	3.97379	0.01878
C	-0.36721	3.048831	0.034776
C	-3.47809	2.551619	-0.1712
C	-0.48274	1.627695	-0.03598
C	-2.9846	1.210125	-0.19845
C	-1.6461	0.819071	-0.13419

H	-3.41313	4.638772	-0.07484
H	-1.07316	5.015299	0.083838
H	0.646726	3.430052	0.108563
H	-4.56162	2.63322	-0.2333
H	-3.72122	0.420429	-0.25911
C	0.25323	-0.49975	-0.12073
C	-1.162	-0.56192	-0.19759
C	2.385547	-1.37824	-0.01428
C	3.244904	-2.51854	-0.01102
N	2.961807	-0.11903	0.066369
C	4.610921	-2.36878	0.064345
H	2.764471	-3.48732	-0.07683
C	4.305871	0.013664	0.140076
C	5.161704	-1.07145	0.1409
H	4.667544	1.033622	0.199497
H	6.231362	-0.91015	0.20058
C	-1.92286	-1.81331	-0.37345
O	-1.52357	-2.7841	-0.98824
N	1.070729	-1.57412	-0.09963
O	-3.13876	-1.76442	0.226841
C	-3.95828	-2.95435	0.094621
C	-5.25072	-2.708	0.849442
H	-3.40137	-3.80493	0.49882
H	-4.12931	-3.14308	-0.9698
H	-5.88933	-3.59478	0.779797
H	-5.05587	-2.50661	1.907023
H	-5.79913	-1.85875	0.430379
H	5.258508	-3.24	0.063304
N	0.6564	0.798811	-0.02192
B	2.112997	1.226276	0.105102
F	2.529991	2.038111	-0.95691
F	2.378574	1.876151	1.317074

S_1 minimum energy structure of **2a**+H+ (B3LYP 6-31g+(d, p))

No imaginary frequency

Zero-point correction = 0.303700 (Hartree)

Thermal correction to Energy = 0.324950

Thermal correction to Enthalpy = 0.325894

Thermal correction to Gibbs Free Energy = 0.252289

Sum of electronic and zero-point Energies = -1195.750429

Sum of electronic and thermal Energies = -1195.729179

Sum of electronic and Enthalpies = -1195.728235

Sum of electronic and thermal Free Energies = -1195.801839

C	-2.85276	3.770723	-6.3E-05
C	-1.42759	3.997351	-0.00002
C	-0.40441	3.096495	-8E-06
C	-3.53585	2.565293	-0.00011
C	-0.543	1.656672	-3.1E-05
C	-3.04872	1.243362	-0.00011
C	-1.68335	0.863566	-7.6E-05
H	-3.46316	4.668154	-6.4E-05
H	-1.13204	5.043216	0.000002
H	0.607814	3.483645	0.000013
H	-4.62058	2.646924	-0.00013
H	-3.77698	0.444878	-0.00014
C	0.228116	-0.4441	-2.6E-05
C	-1.20278	-0.53687	-6.6E-05
C	2.460008	-1.35253	-2.6E-05
C	3.295737	-2.4826	-8.1E-05
N	2.973317	-0.09445	-2E-06
C	4.667979	-2.30001	-0.00012

H	2.852779	-3.4725	-9.9E-05
C	4.321366	0.073175	-4.8E-05
C	5.194277	-0.99738	-0.00011
H	4.660004	1.102267	-3.4E-05
H	6.262566	-0.81798	-0.00014
C	-1.87333	-1.84359	-8.4E-05
O	-1.21629	-2.88859	-2E-06
N	1.09588	-1.49013	-1.3E-05
O	-3.1966	-1.79117	-4.6E-05
C	-3.91691	-3.0749	0.00002
C	-5.39805	-2.76752	0.000028
H	-3.59915	-3.62873	0.887217
H	-3.59918	-3.62881	-0.88714
H	-5.95512	-3.70932	0.000077
H	-5.68749	-2.20269	0.890604
H	-5.68751	-2.20277	-0.89059
H	5.328696	-3.16055	-0.00016
N	0.615621	0.824969	-1.2E-05
B	2.10281	1.272113	0.000176
F	2.41552	1.968862	-1.14663
F	2.415333	1.968449	1.147282
H	0.642581	-2.40952	-3.9E-05

S_0 minimum energy structure of **2b** (B3LYP 6-31g+(d, p))

No imaginary frequency

Zero-point correction = 0.294782 (Hartree)

Thermal correction to Energy = 0.318330

Thermal correction to Enthalpy = 0.319274

Thermal correction to Gibbs Free Energy = 0.239142

Sum of electronic and zero-point Energies = -1399.944808

Sum of electronic and thermal Energies = -1399.921261

Sum of electronic and Enthalpies = -1399.920316

Sum of electronic and thermal Free Energies = -1400.000448

C	4.22953	-3.00191	0.008464
C	2.996943	-3.63367	0.079243
C	1.710305	-3.06968	0.074071
C	4.501953	-1.62153	-0.08611
C	1.365759	-1.72764	-0.00089
C	3.632775	-0.54503	-0.12935
C	2.217736	-0.53877	-0.09218
H	5.100255	-3.65257	0.02807
H	3.021054	-4.7183	0.147658
H	0.881647	-3.76692	0.136791
H	5.557118	-1.36153	-0.12937
H	4.087219	0.43654	-0.19231
C	0.017282	0.075124	-0.08103
C	1.356777	0.572695	-0.15672
C	-2.28044	0.172475	0.012207
C	-3.49184	0.934433	0.044246
N	-2.43608	-1.20129	-0.00764
C	-4.73185	0.349111	-0.03723
C	-3.66186	-1.78448	-0.05066
C	-4.82697	-1.05483	-0.08099
H	-3.65565	-2.86799	-0.06469
H	-5.78584	-1.55519	-0.12693
C	1.674318	2.003751	-0.32944
O	0.920172	2.831058	-0.79739
N	-1.10197	0.786566	-0.04916
O	2.934523	2.311453	0.091982
C	3.319212	3.703516	-0.0485
C	4.735718	3.845222	0.475935
H	2.609712	4.316573	0.514548
H	3.23891	3.984383	-1.10296

H	5.051396	4.890805	0.398183
H	4.798558	3.545997	1.526659
H	5.437216	3.235397	-0.10256
H	-5.6163	0.97677	-0.05516
N	0.060855	-1.30633	0.003121
B	-1.20119	-2.18944	0.061107
F	-1.26104	-3.06331	-1.02665
F	-1.26768	-2.90893	1.255861
N	-3.43258	2.397906	0.17738
O	-4.11052	3.052314	-0.61858
O	-2.76723	2.858004	1.09666

S_0 minimum energy structure of **2b**+H^+ (B3LYP 6-31g+(d, p))

No imaginary frequency

Zero-point correction = 0.308829 (Hartree)

Thermal correction to Energy = 0.332171

Thermal correction to Enthalpy = 0.333115

Thermal correction to Gibbs Free Energy = 0.254045

Sum of electronic and zero-point Energies = -1400.338107

Sum of electronic and thermal Energies = -1400.314765

Sum of electronic and Enthalpies = -1400.313821

Sum of electronic and thermal Free Energies = -1400.392892

C	4.351135	-2.92784	-0.00006
C	3.131127	-3.6014	-7.2E-05
C	1.835821	-3.07251	-5.5E-05
C	4.598307	-1.54491	-2.6E-05

C	1.470337	-1.73098	-1.5E-05
C	3.698974	-0.48523	0.000011
C	2.292477	-0.53201	0.000022
H	5.235514	-3.55975	-8.1E-05
H	3.187491	-4.68628	-0.0001
H	1.022693	-3.79166	-8.5E-05
H	5.647242	-1.26066	-2.5E-05
H	4.125073	0.511922	0.000039
C	0.10667	0.020266	0.000058
C	1.396234	0.574439	0.000071
C	-2.31122	0.116741	-5E-06
C	-3.52829	0.853364	-6.1E-05
N	-2.3854	-1.24338	-0.00006
C	-4.7467	0.191224	-0.00018
C	-3.58202	-1.88346	-0.00018
C	-4.78167	-1.20449	-0.00025
H	-3.52482	-2.96549	-0.00023
H	-5.71837	-1.74739	-0.00035
C	1.603501	2.031474	0.000123
O	0.656746	2.809712	0.000057
N	-1.0791	0.69747	0.000088
O	2.884282	2.406458	0.000025
C	3.145212	3.853329	-4.9E-05
C	4.646435	4.046926	-0.00016
H	2.667107	4.276664	0.886457
H	2.66698	4.276598	-0.88652
H	4.867677	5.118591	-0.00022
H	5.105298	3.607939	0.890693
H	5.10517	3.607873	-0.89105
H	-5.65396	0.78476	-0.00023
N	0.144893	-1.33509	0.000002
B	-1.11007	-2.24009	0.000142
F	-1.17679	-3.00606	-1.1452
F	-1.17683	-3.00564	1.145762
H	-1.01648	1.726109	0.000136

N	-3.56005	2.326568	-2E-06
O	-4.65887	2.86338	-9.4E-05
O	-2.48017	2.927686	0.000092

S_1 minimum energy structure of **2b** (B3LYP 6-31g+(d, p))

No imaginary frequency

Zero-point correction = 0.291675 (Hartree)

Thermal correction to Energy = 0.315696

Thermal correction to Enthalpy = 0.316641

Thermal correction to Gibbs Free Energy = 0.235380

Sum of electronic and zero-point Energies = -1399.860380

Sum of electronic and thermal Energies = -1399.836359

Sum of electronic and Enthalpies = -1399.835414

Sum of electronic and thermal Free Energies = -1399.916675

C	4.421643	-2.8198	-0.07283
C	3.176157	-3.54869	0.059366
C	1.902208	-3.063	0.078607
C	4.621557	-1.46582	-0.20505
C	1.494946	-1.68914	-0.02726
C	3.678842	-0.40208	-0.24871
C	2.284451	-0.52972	-0.16609
H	5.316675	-3.43644	-0.06493
H	3.278714	-4.62624	0.153251
H	1.098201	-3.78444	0.182726
H	5.660576	-1.15239	-0.2873
H	4.072787	0.602251	-0.33705
C	0.036057	0.025778	-0.13768
C	1.338724	0.586253	-0.24846

N	-1.10522	0.737715	-0.10517
C	-2.2564	0.095242	0.012946
C	-3.49443	0.818238	0.033146
N	-2.35359	-1.29019	0.065806
C	-4.71448	0.18533	0.014538
C	-3.55451	-1.91016	0.08324
C	-4.75059	-1.22054	0.046893
H	-5.62353	0.775993	-0.01305
H	-3.51458	-2.99253	0.122421
H	-5.68934	-1.76	0.052729
C	1.605532	2.017809	-0.49388
O	0.957426	2.696214	-1.26668
O	2.65694	2.476621	0.217418
C	2.981367	3.883899	0.037794
C	4.124762	4.213426	0.977204
H	2.084995	4.47211	0.253484
H	3.245316	4.045197	-1.01192
H	4.389363	5.270555	0.871324
H	3.841968	4.032472	2.018321
H	5.011565	3.614304	0.748985
N	-3.48339	2.287397	0.082925
O	-2.80284	2.822196	0.949675
O	-4.20896	2.869401	-0.72622
N	0.136092	-1.32516	-0.00657
B	-1.07351	-2.24351	0.146449
F	-1.16495	-3.18077	-0.88528
F	-1.09705	-2.89582	1.381239

S_1 minimum energy structure of **2b**+H^+ Structure A (B3LYP 6-31g+(d, p))

No imaginary frequency

Zero-point correction = 0.305473 (Hartree)

Thermal correction to Energy = 0.329321

Thermal correction to Enthalpy = 0.330265

Thermal correction to Gibbs Free Energy = 0.250052

Sum of electronic and zero-point Energies = -1400.236470

Sum of electronic and thermal Energies = -1400.212622

Sum of electronic and Enthalpies = -1400.211678

Sum of electronic and thermal Free Energies = -1400.291891

C	4.416605	-2.89496	0.000231
C	3.158175	-3.58955	0.00018
C	1.884811	-3.09338	0.000129
C	4.645519	-1.52361	0.000246
C	1.525687	-1.69419	0.000115
C	3.739025	-0.45131	0.000215
C	2.32192	-0.5581	0.000153
H	5.297501	-3.52857	0.000265
H	3.235399	-4.67382	0.000181
H	1.067933	-3.80501	0.000093
H	5.692757	-1.22944	0.000294
H	4.150096	0.548063	0.000242
C	0.072508	0.018113	0.000037
C	1.393598	0.591268	0.00012
C	-2.33369	0.101438	-0.00009
C	-3.5597	0.822687	-0.00012
N	-2.37957	-1.26163	-0.00014
C	-4.76731	0.142629	-0.00022
C	-3.56562	-1.91786	-0.00024
C	-4.7765	-1.25477	-0.00029
H	-3.49464	-2.99927	-0.00028
H	-5.70428	-1.81312	-0.00039
C	1.593173	2.048893	0.000117

O	0.626069	2.807967	-4.8E-05
N	-1.10744	0.69639	-9E-06
O	2.860934	2.435873	0.000116
C	3.10873	3.888287	0.000009
C	4.606745	4.096826	0.000031
H	2.622341	4.30276	0.886797
H	2.622387	4.302624	-0.88687
H	4.813961	5.171226	-4.4E-05
H	5.069707	3.663095	0.890884
H	5.069752	3.662962	-0.89074
H	-5.68518	0.719777	-0.00023
N	0.153566	-1.30602	0.000046
B	-1.08159	-2.241	-4.6E-05
F	-1.13722	-2.99831	-1.14733
F	-1.13739	-2.99831	1.14723
H	-1.06241	1.725753	-3.9E-05
N	-3.60663	2.294638	-4.7E-05
O	-4.70924	2.822102	-4.5E-05
O	-2.52997	2.905229	-0.00024

S_1 minimum energy structure of **2b**+H^+ Structure B (B3LYP 6-31g+(d, p))

No imaginary frequency

Zero-point correction = 0.304453 (Hartree)

Thermal correction to Energy = 0.328149

Thermal correction to Enthalpy = 0.329093

Thermal correction to Gibbs Free Energy = 0.249287

Sum of electronic and zero-point Energies = -1400.251705

Sum of electronic and thermal Energies = -1400.228009

Sum of electronic and Enthalpies = -1400.227065

Sum of electronic and thermal Free Energies = -1400.306871

C	4.1969	-3.05984	-0.00187
C	2.941723	-3.68102	-0.00139
C	1.666092	-3.10643	-0.00083
C	4.498248	-1.69168	-0.00184
C	1.35038	-1.75174	-0.00055
C	3.637378	-0.59635	-0.00122
C	2.224933	-0.58531	-0.00057
H	5.051689	-3.73079	-0.0023
H	2.955684	-4.76755	-0.00153
H	0.826485	-3.79338	-0.00071
H	5.556706	-1.44586	-0.00228
H	4.101342	0.382856	-0.00113
C	0.009443	0.044364	0.000617
C	1.384512	0.549454	0.000192
C	-2.32274	0.12792	0.000177
C	-3.4818	0.952515	-0.00126
N	-2.46691	-1.19925	0.000411
C	-4.75809	0.395013	-0.00186
C	-3.74461	-1.78635	-0.00032
C	-4.86735	-1.01797	-0.00135
H	-3.75027	-2.86727	0.00008
H	-5.8426	-1.48953	-0.00171
C	1.685732	1.996056	-0.00033
O	0.808052	2.842452	-0.00208
N	-1.10094	0.757034	0.001629
O	2.997265	2.268359	0.001182
C	3.365159	3.691861	0.000751
C	4.876237	3.775964	0.003101
H	2.916618	4.149601	0.885925
H	2.919435	4.148349	-0.88649
H	5.17379	4.828918	0.002864
H	5.3012	3.306702	0.895394
H	5.304053	3.305526	-0.88721

H	-5.62932	1.035795	-0.00275
N	0.038467	-1.31303	0.000042
B	-1.25081	-2.20499	0.002027
F	-1.28757	-2.98139	-1.14247
F	-1.28684	-2.97723	1.149305
H	-1.44684	2.220647	-0.00361
N	-3.35552	2.382942	0.000036
O	-4.35485	3.074069	0.00382
O	-2.17458	2.994588	-0.00665

S_0 minimum energy structure of **2c** (B3LYP 6-31g+(d, p))

No imaginary frequency

Zero-point correction = 0.295069 (Hartree)

Thermal correction to Energy = 0.318485

Thermal correction to Enthalpy = 0.319429

Thermal correction to Gibbs Free Energy = 0.238791

Sum of electronic and zero-point Energies = -1399.958154

Sum of electronic and thermal Energies = -1399.934738

Sum of electronic and Enthalpies = -1399.933794

Sum of electronic and thermal Free Energies = -1400.014432

C	3.734916	3.59569	-0.01044
C	2.375625	3.877294	0.005327
C	1.290112	2.989274	0.003296
C	4.364496	2.336619	-0.03165
C	1.3179	1.599848	-0.01327

C	3.815266	1.06384	-0.04015
C	2.45465	0.677394	-0.03184
H	4.400235	4.455423	-0.00614
H	2.1068	4.930399	0.02056
H	0.30362	3.440034	0.016195
H	5.451678	2.364872	-0.04178
H	4.518561	0.241066	-0.05224
C	0.49044	-0.49453	-0.03574
C	1.916528	-0.62882	-0.04997
N	0.170093	0.852011	-0.01382
N	-0.41175	-1.47053	-0.03001
C	-1.7138	-1.18484	-0.01226
C	-2.65059	-2.26365	-0.00837
C	-3.99964	-2.03128	0.006134
H	-2.23647	-3.26421	-0.01946
C	-3.54466	0.344973	0.015009
C	-4.45102	-0.69033	0.017647
H	-4.72261	-2.83798	0.008257
H	-3.86311	1.379833	0.024442
N	-2.21438	0.109092	0.0011
C	2.598169	-1.93591	-0.09291
O	2.049929	-3.00783	-0.253
O	3.949795	-1.82854	0.069167
C	4.687102	-3.07692	0.035082
C	6.157213	-2.7492	0.217618
H	4.491166	-3.57261	-0.92022
H	4.309482	-3.72555	0.830925
H	6.742404	-3.67452	0.204479
H	6.523129	-2.10513	-0.58841
H	6.334746	-2.24746	1.174019
N	-5.87407	-0.38841	0.03287
O	-6.21689	0.797169	0.041584
O	-6.6539	-1.34523	0.036214
B	-1.27962	1.385044	0.008261
F	-1.52915	2.121826	1.165823

F	-1.55291	2.153086	-1.12313

S_0 minimum energy structure of **2c**+H^+ (B3LYP 6-31g+(d, p))

No imaginary frequency

Zero-point correction = 0.308887 (Hartree)

Thermal correction to Energy = 0.332313

Thermal correction to Enthalpy = 0.333258

Thermal correction to Gibbs Free Energy = 0.253906

Sum of electronic and zero-point Energies = -1400.339691

Sum of electronic and thermal Energies = -1400.316264

Sum of electronic and Enthalpies = -1400.315320

Sum of electronic and thermal Free Energies = -1400.394671

C	3.811054	3.625306	-0.00011
C	2.451273	3.93088	-9.8E-05
C	1.354914	3.061458	-7.5E-05
C	4.435154	2.366673	-0.00014
C	1.378653	1.671277	-8.1E-05
C	3.867752	1.097727	-0.00016
C	2.504607	0.750367	-7.4E-05
H	4.483421	4.479287	-0.00011
H	2.202184	4.988302	-0.00011
H	0.373107	3.524698	-8.2E-05
H	5.521735	2.387145	-0.00018
H	4.556135	0.259602	-0.0002
C	0.560073	-0.38731	-7.2E-05
C	1.952057	-0.56265	-4.1E-05
N	0.214266	0.921657	-0.0001
N	-0.38314	-1.3707	-0.00011

C	-1.73418	-1.16033	-6.2E-05
C	-2.62863	-2.24783	-0.00013
C	-3.98697	-2.00552	-0.00013
H	-2.23727	-3.25884	-0.00018
C	-3.52376	0.363741	-1.5E-05
C	-4.42713	-0.6738	-7.5E-05
H	-4.71171	-2.81192	-0.00017
H	-3.83826	1.400277	0.000019
N	-2.18994	0.120362	0.000006
C	2.53929	-1.9082	0.000014
O	1.829748	-2.9151	0.00007
O	3.869673	-1.9351	0.000014
C	4.5099	-3.25968	0.000095
C	6.007697	-3.0425	0.000078
H	4.162929	-3.7948	-0.88698
H	4.162933	-3.79468	0.887244
H	6.508476	-4.01544	0.000122
H	6.331433	-2.49654	-0.89084
H	6.331444	-2.49646	0.890946
N	-5.86912	-0.36703	-7.9E-05
O	-6.18748	0.818047	-5.2E-05
O	-6.63414	-1.32617	-5.7E-05
B	-1.2524	1.440826	0.000222
F	-1.52547	2.153003	1.146865
F	-1.52579	2.153613	-1.14597
H	0.0192	-2.31544	-4.3E-05

S_1 minimum energy structure of **2c** (B3LYP 6-31g+(d, p))

No imaginary frequency

Zero-point correction = 0.291885 (Hartree)

Thermal correction to Energy = 0.315732

Thermal correction to Enthalpy = 0.316677

Thermal correction to Gibbs Free Energy = 0.235937

Sum of electronic and zero-point Energies = -1399.872642

Sum of electronic and thermal Energies = -1400.848795

Sum of electronic and Enthalpies = -1400.847850

Sum of electronic and thermal Free Energies = -1400.928590

C	3.838135	3.580173	-0.04876
C	2.430364	3.906025	0.037907
C	1.358013	3.064226	0.039507
C	4.434589	2.343415	-0.1467
C	1.381529	1.628281	-0.04436
C	3.853569	1.048814	-0.18784
C	2.481016	0.753238	-0.13822
H	4.509222	4.434917	-0.03424
H	2.204803	4.966499	0.109052
H	0.373477	3.515021	0.108657
H	5.521663	2.352255	-0.19906
H	4.530886	0.207216	-0.24739
C	0.495082	-0.44047	-0.15646
C	1.909462	-0.59521	-0.21832
N	0.191913	0.881287	-0.04904
N	-0.39328	-1.45428	-0.15369
C	-1.68924	-1.17757	-0.07806
C	-2.62649	-2.25692	-0.09353
C	-3.97641	-2.02401	-0.02936
H	-2.21531	-3.25642	-0.16463
C	-3.50368	0.354677	0.072699
C	-4.41557	-0.68411	0.054037
H	-4.70315	-2.8269	-0.04276
H	-3.82398	1.387193	0.139204

N	-2.18015	0.124041	0.010452
C	2.59179	-1.891	-0.40431
O	2.155466	-2.79347	-1.09301
O	3.763381	-1.95683	0.266676
C	4.504756	-3.20096	0.13339
C	5.746888	-3.08924	0.995612
H	4.743982	-3.34834	-0.92419
H	3.855431	-4.02299	0.448167
H	6.323785	-4.01705	0.924636
H	6.385826	-2.26415	0.666489
H	5.483904	-2.92742	2.04507
N	-5.83756	-0.37688	0.123629
O	-6.17134	0.809052	0.191754
O	-6.62001	-1.33118	0.109945
B	-1.23781	1.412997	0.072764
F	-1.45995	2.05428	1.291096
F	-1.58126	2.254516	-0.98428

S_1 minimum energy structure of **2c**+H^+ (B3LYP 6-31g+(d, p))

No imaginary frequency

Zero-point correction = 0.305437 (Hartree)

Thermal correction to Energy = 0.329377

Thermal correction to Enthalpy = 0.330321

Thermal correction to Gibbs Free Energy = 0.249839

Sum of electronic and zero-point Energies = -1400.236756

Sum of electronic and thermal Energies = -1400.212816

Sum of electronic and Enthalpies = -1400.211872

Sum of electronic and thermal Free Energies = -1400.292354

C	3.852636	3.627413	-0.00015
C	2.447918	3.934835	-0.00016
C	1.368639	3.096246	-0.00011
C	4.464312	2.379661	-8.4E-05
C	1.422716	1.653444	-3.6E-05
C	3.900489	1.092741	-1.4E-05
C	2.512975	0.791793	0.000008
H	4.515615	4.486514	-0.00019
H	2.213257	4.996165	-0.00021
H	0.382433	3.545076	-0.00014
H	5.551884	2.3969	-0.00009
H	4.57986	0.251974	0.000029
C	0.523633	-0.39823	0.000085
C	1.951191	-0.57386	0.000087
N	0.217406	0.891404	0.000009
N	-0.4062	-1.38936	0.000148
C	-1.75765	-1.17462	0.000096
C	-2.65962	-2.25436	0.000117
C	-4.0171	-1.99922	0.000046
H	-2.27801	-3.26935	0.000185
C	-3.52533	0.369612	-6.5E-05
C	-4.4399	-0.66152	-0.00005
H	-4.75096	-2.79711	0.000059
H	-3.83278	1.408558	-0.00014
N	-2.1953	0.11585	0.000013
C	2.542191	-1.91951	0.000155
O	1.818088	-2.91924	0.000131
O	3.864146	-1.94783	0.000063
C	4.506939	-3.27481	0.000027
C	6.003528	-3.05763	-7.6E-05
H	4.154834	-3.80693	-0.88742
H	4.154954	-3.80692	0.887529
H	6.50144	-4.03197	-0.00011
H	6.327203	-2.5123	-0.89098
H	6.327324	-2.51229	0.890773

N	-5.87843	-0.33794	-0.00014
O	-6.18247	0.851048	-0.00026
O	-6.65326	-1.28933	-0.00013
B	-1.2424	1.433219	0.000069
F	-1.5082	2.139427	1.148179
F	-1.50826	2.139566	-1.14794
H	-0.00669	-2.33409	0.000211

Experimental section

Materials

Tropolone and phosphorus (V) oxychloride were purchased from Tokyo Chemical Industry (Tokyo, Japan). Sodium was purchased from Kanto chemical. Ammonia solution (28% in water), 6 mol/L hydrochloric acid, magnesium sulfate, and sodium carbonate, were purchased from FUJIFILM Wako Chemical Corp. (Osaka, Japan). Diethyl malonate was purchased from Sigma-Aldrich (Tokyo, Japan).

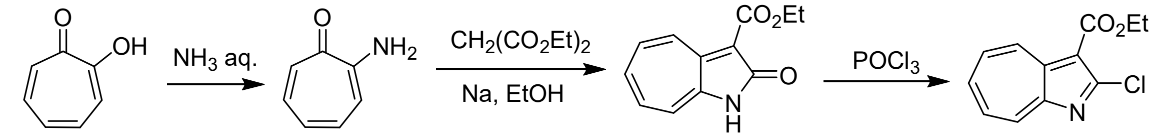

4 **5** **6** **7**

Preparation of 2-aminotropone (**5**)

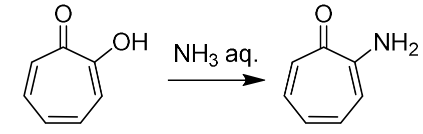

4 **5**

In Young's tube, tropolone (5.00 g, 40.9 mmol) was added 28% ammonia aqueous solution (20 mL) and heating at 170°C for 1 h with stirring. The solution was extracted with chloroform; the organic layer was washing with water and dried over $MgSO_4$, filtration, and evaporation. Yellowish-brown powders were obtained (4.34 g, 87%).

5: Yellowish-brown powder; m.p. 105.5-106.4°C, yield: 87% (lit. [1)] m.p. 106-107°C, yield: 99%)

^{1}H NMR ($CDCl_3$/400 MHz): δ (ppm) 7.25-7.30 (m, 1H), 7.22 (d, *J*=10.4 Hz, 1H), 7.15 (t, *J*=10.4 Hz, 1H), 6.91 (d, *J*=10.0 Hz, 1H), 6.74 (t, *J*=9.4 Hz, 1H), 6.12 (brs, 2H).

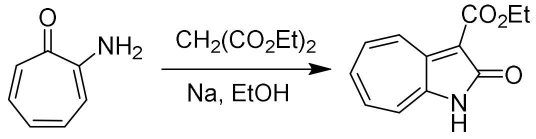

5 **6**

After preparation of sodium ethoxide from ethanol (60 mL) and sodium (0.90 g, 40 mmol), **5** (2.00 g, 16.5 mmol) and diethyl malonate (5.2mL. 34 mmol) were added and reflux for 48 h. The solution was cooling in ice-bath; the precipitation, which was formed after standing for overnight, was filtration. The solid was dispersed into water (40 mL). After addition of hydrochloric acid and cooling in ice-bath, the yellow powder was obtained. The yellow powder was purified by recrystallization with ethyl acetate and ethanol; the orange crystals were obtained (1.90 g, 53%).

Orange crystal; m.p. 191.2-192.2°C yield: 53% (lit. [2)] 189-190°C, yield: 53%)

^{1}H NMR ($CDCl_3$ /400 MHz): δ (ppm) 13.07 (brs, 1H), 9.07 (d, *J*=11.2 Hz, 1H), 7.86 (d, *J*=9.6 Hz, 1H), 7.65 (t, *J*=9.8 Hz, 1H), 7.59 (t, *J*=9.8 Hz, 1H), 7.41 (t, *J*=9.2 Hz, 1H), 4.48 (q, *J*=6.8 Hz, 2H), 1.47 (t, *J*=6.8 Hz, 3H).

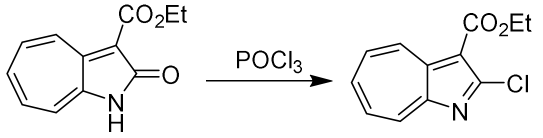

6 **7**

6 (1.00 g, 4.60 mmol) was added $POCl_3$ (3.3 mL, 36 mmol) and heating at 110°C for 1h with stirring. The solution was diluted with ice-water, neutralized with Na_2CO_3aq., and extracted with chloroform. The organic layer was washing with water and dried over $MgSO_4$, filtration, and evaporation. The solid was purified by silica gel column chromatography (chloroform): the orange needles were obtained (0.94 g, 87%).

Orange needle crystals, m.p. 82.8-83.1°C, yield: 87% (lit.[3)] 84.5-85.5°C, yield: 73%)
^{1}H NMR ($CDCl_3$ /400 MHz): δ (ppm) 9.58 (d, *J*=10.0 Hz, 1H), 8.71 (d, *J*=9.6 Hz, 1H), 8.07 (t, *J*=9.6 Hz, 1H), 7.98 (t, *J*=10.0 Hz, 1H), 7.98 (t, *J*=10.0 Hz, 1H), 4.49 (q, *J*=7.2 Hz, 2H), 1.48 (t, *J*=7.2 Hz, 3H).

2.9 Preparation of PS film

1 mg/mL chloroform solution of **2** and 0.1 g/mL chloroform solution of poly(styrene) were prepared and mixed in the amounts shown in Table S11. The solution was dropped in the petri dish (Φ=3 cm) and evaporated under r. t.

Table S11. Preparation of PS film

wt%	**2** solution (mL)	PS solution (mL)	Chloroform (mL)
0.1	0.1 mL	1 mL	2.9 mL
1	1 mL	1 mL	2 mL
3	3 mL	1 mL	0 mL

2.10 Preparation of PS film addition of acid

0.1 mg/mL chloroform solution of **2**, 0.1 g/mL chloroform solution of poly(styrene), 0.55 mg/mL and 5.5 mg/mL chloroform solution of *p*-toluenesulfonic acid were prepared and mixed in the amounts shown in Table S12. The solution was dropped in the petri dish (Φ=3 cm) and evaporated under r. t.

Table S12. Preparation of PS added *p*-toluenesulfonic acid

acid equivalent	**2** solution (mL)	PS solution (mL)	dilute acid solution (mL)	concentrated acid solution (mL)	chloroform (mL)
1	1	1	0.1	-	1.9
3	1	1	0.3	-	1.7
5	1	1	0.5	-	1.5
10	1	1	1	-	1
20	1	1	-	0.2	1.8
50	1	1	-	0.5	1.5
100	1	1	-	1	1

References

1) Kubo, K.; Mori, A. Japan Patent JP4202148B2.

2) Nagahara, M.; Nakao, J.; Mimura, M.; Nakamura, T.; Uchida, K.; Synthesis and Antiallergic Activity of Novel Azaazulene Derivatives. *Chem. Pharm. Bull.*, **1994**, *42*, 2491–2499. https://doi.org/10.1248/cpb.42.2491

3) Tsukada, S.; Nakazawa, M.; Okada, Y.; Ohtsu, K.; Abe, N.; Gunji, T. SYNTHESIS OF 2-ARYLAMINO-1-AZAAZULENES. *Heterocycles*, **2017**, *95*, 624–635. DOI: 10.3987/COM-16-S(S)48
